# Liquid biopsy posttreatment surveillance in endemic nasopharyngeal carcinoma: a cost-effective strategy to integrate circulating cell-free Epstein-Barr virus DNA

**DOI:** 10.1186/s12916-021-02076-4

**Published:** 2021-08-26

**Authors:** Chen-Fei Wu, Li Lin, Yan-Ping Mao, Bin Deng, Jia-Wei Lv, Wei-Hong Zheng, Dan-Wan Wen, Jia Kou, Fo-Ping Chen, Xing-Li Yang, Si-Si Xu, Jun Ma, Guan-Qun Zhou, Ying Sun

**Affiliations:** 1 Department of Radiation Oncology, Sun Yat-sen University Cancer Center; State Key Laboratory of Oncology in South China, Collaborative Innovation Center for Cancer Medicine, Guangdong Key Laboratory of Nasopharyngeal Carcinoma Diagnosis and Therapy, 651 Dongfeng Road East, Guangzhou, 510060 Guangdong People’s Republic of China; 2 Department of Radiation Oncology, Wuzhou Red Cross Hospital, Wuzhou, 543002 Guangxi People’s Republic of China

**Keywords:** Liquid biopsy, Circulating cell-free Epstein-Barr virus DNA, Posttreatment surveillance, Surveillance imaging, Cost-effectiveness, Nasopharyngeal carcinoma

## Abstract

**Background:**

The optimal posttreatment surveillance strategy for nasopharyngeal carcinoma (NPC) remains unclear. Circulating cell-free Epstein-Barr virus (cfEBV) DNA has been recognized as a promising biomarker to facilitate early detection of NPC recurrence. Therefore, we aim to determine whether integrating circulating cfEBV DNA into NPC follow-up is cost-effective.

**Methods:**

For each stage of asymptomatic nonmetastatic NPC patients after complete remission to primary NPC treatment, we developed a Markov model to compare the cost-effectiveness of the following surveillance strategies: routine follow-up strategy, i.e., (1) routine clinical physical examination; routine imaging strategies, including (2) routine magnetic resonance imaging plus computed tomography plus bone scintigraphy (MRI + CT + BS); and (3) routine ^18^F-fluorodeoxyglucose positron emission tomography/computed tomography (PET/CT); cfEBV DNA-guided imaging strategies, including (4) cfEBV DNA-guided MRI + CT + BS and (5) cfEBV DNA-guided PET/CT. Clinical probabilities, utilities, and costs were derived from published studies or databases. Sensitivity analyses were performed.

**Results:**

For all disease stages, cfEBV DNA-guided imaging strategies demonstrated similar survival benefits but were considerably more economical than routine imaging strategies. They only required approximately one quarter of the number of imaging studies compared with routine imaging strategies to detect one recurrence. Specifically, cfEBV DNA-guided MRI + CT + BS was most cost-effective for stage II (incremental cost-effectiveness ratio [ICER] $57,308/quality-adjusted life-year [QALY]) and stage III ($46,860/QALY) patients, while cfEBV DNA-guided PET/CT was most cost-effective for stage IV patients ($62,269/QALY). However, routine follow-up was adequate for stage I patients due to their low recurrence risk.

**Conclusions:**

The cfEBV DNA-guided imaging strategies are effective and cost-effective follow-up methods in NPC. These liquid biopsy-based strategies offer evidence-based, stage-specific surveillance modalities for clinicians and reduce disease burden for patients.

**Supplementary Information:**

The online version contains supplementary material available at 10.1186/s12916-021-02076-4.

## Background

More than 20% of patients with nonmetastatic nasopharyngeal carcinoma (NPC) will experience disease recurrence despite aggressive primary treatment, contributing largely to treatment failure and death [1, 2]. The posttreatment surveillance is essential to detect recurrence timely when the tumor burden is minimal, which would maximize the efficacy of salvage treatment and might improve survival [3–6]. However, surveillance can be challenging in NPC, and there is no consensus on the optimal follow-up modalities. Consequently, practice varies widely across clinicians and institutions. Although routine surveillance imaging, e.g., routine magnetic resonance imaging (MRI) and computed tomography (CT), is efficacious in detecting recurrence, it has been questioned due to the prohibitive resource consumption and economic burden [7]. Hence, it is imperative to identify a follow-up strategy that can detect recurrence in a timely and cost-effective manner.

Epstein-Barr virus (EBV) infections were predominant in endemic NPC [8]. Circulating cell-free EBV (cfEBV) DNA, short DNA fragments released by NPC cells, has been established as an NPC biomarker in population screening [9], risk assessment [8, 10], treatment evaluation [11, 12], and follow-up [13–16]. Several studies have shown that the posttreatment cfEBV DNA tests could facilitate early detection of NPC recurrence, especially for distant recurrence [13–18]. Specifically, detectable plasma cfEBV DNA during follow-up indicates tumor recurrence, while undetectable cfEBV DNA demonstrates continuous remission [15, 16, 18]. A recent work involving 1984 nonmetastatic NPC patients demonstrated that the sensitivity and specificity for cfEBV DNA to detect recurrence were up to 82.3 and 80.0%, respectively, highlighting the efficacy of cfEBV DNA surveillance [16]. Another study investigating cfEBV DNA surveillance followed by positron emission tomography/computed tomography (PET/CT) indicated that such strategy could correctly identify NPC recurrence while saved approximately four-fifths in expenses [14]. Therefore, the updated European Society for Medical Oncology (ESMO) guideline for NPC has recognized cfEBV DNA as a promising biomarker for recurrence and recommended evaluating it at least every year [17], and the National Comprehensive Cancer Network (NCCN) guidelines also suggested EBV DNA monitoring for NPC [19]. However, little evidence is available regarding how to integrate cfEBV DNA into the current surveillance series [14, 17].

Considering that cfEBV DNA could promptly suggest disease recurrence with high accuracy [14–16], we proposed a liquid biopsy-based stepwise surveillance strategy, starting with routine cfEBV DNA tests, followed by further imaging studies if the tests are positive. We estimated its cost-effectiveness and compared it with other surveillance strategies in each stage of NPC. We speculated that selected imaging studies for patients with positive cfEBV DNA was a cost-effective follow-up modality, demonstrating the potential application of circulating cell-free DNA in the follow-up of cancer survivors. We aimed to establish evidence-based, stage-specific NPC surveillance strategies and investigate the potential application of circulating cell-free DNA in the follow-up of cancer survivors.

## Methods

### Model construction

We developed a Markov decision-analytic model to evaluate the cost-effectiveness of five surveillance strategies in NPC (Additional file 1: Fig. S1A): routine follow-up strategy, i.e., (1) routine clinical physical examination; routine imaging strategies, including (2) routine MRI plus CT plus bone scintigraphy (MRI + CT + BS); and (3) routine ^18^F-fluorodeoxyglucose PET/CT; cfEBV DNA-guided imaging strategies, including (4) cfEBV DNA-guided MRI + CT + BS; and (5) cfEBV DNA-guided PET/CT. The routine follow-up included history and physical examinations, complete blood counts, comprehensive metabolic panels, and nasopharyngoscopies. The routine imaging strategies included routine follow-up and imaging studies. The cfEBV DNA-guided imaging strategies included routine follow-up and cfEBV DNA tests, whose positive results would trigger further imaging studies. MRI imaged the head and neck; CT imaged the chest and abdomen; BS imaged the whole body; and PET/CT imaged from the skull base to midthigh. According to the previous study [20], the surveillance was scheduled as follows: (1) stage I: 2 follow-up visits in year 1; 3 visits in year 2; 2 visits in year 3 and 4; and 1 visit in year 5, (2) stage II: 2 visits in year 1; 4 visits in year 2; 2 visits in year 3 and 4; and 1 visit in year 5, (3) stage III: 4 visits in year 1 and 2; 3 visits in year 3; and 1 visit in year 4 and 5, and (4) stage IV: 4 visits in year 1; 5 visits in year 2; 3 visits in year 3; and 1 visit in year 4 and 5. We chose a 5-year follow-up duration because more than 90% of recurrence happened within this interval [21, 22].

We simulated the natural history of four hypothetical cohorts of 45-year-old asymptomatic nonmetastatic NPC patients with stage I, II, III, and IV disease after complete remission (CR) to the primary treatment. In each cohort, patients would move through the following health states: no evidence of disease (NED) after primary treatment, early- and advanced-stage recurrence (local relapse [LR], regional relapse [RR], or distant metastasis [DM]), salvage treatment for recurrence, NED after salvage treatment, and death (Additional file 1: Fig. S1B). In each 1-month cycle, the Markov model would accrue the costs and utilities as patients experienced different health states or events in a lifetime horizon. All future costs and utilities were discounted at a standard rate of 3% annually [23].

The primary outcomes included the cost, effectiveness, incremental cost-effectiveness ratio (ICER), and net health benefit (NHB) of each strategy. The ICER was defined as the incremental cost for an additional quality-adjusted life-year (QALY) gained by comparing two strategies. The willingness-to-pay threshold was set at $100,000/QALY [24, 25]. Strategies with ICERs less than this threshold were considered cost-effective. The NHB, which is explicit and robust when comparing multiple strategies, was calculated with the formula: effectiveness–cost/willingness-to-pay [26]. The strategy with the highest NHB was considered the most cost-effective at the given willingness-to-pay threshold. Additionally, we compared the average number of imaging studies required to detect one disease recurrence among the different surveillance strategies.

All analyses were conducted and reported in line with the recommendations of the Second Panel on Cost-Effectiveness in Health and Medicine and the Consolidated Health Economic Evaluation Reporting Standards [23, 27]. The institutional ethics committee approved the study and waived the requirement for informed consent given its retrospective nature (B2020-410). Tests were two-sided, and *P* < 0.05 was defined as statistically significant. The Markov model was constructed in TreeAge Pro (TreeAge Software, Williamstown, MA), and other statistical analyses were performed using R (version 3.6.1, http://www.r-project.org).

### Model clinical estimates

The base-case estimates are presented in Additional file 1: Table S1 [3, 4, 16, 20, 23, 28–56]. Monthly LR, RR, and DM probabilities were fitted by parametric survival models using the patient-level real-world data of an NPC cohort containing 10,097 nonmetastatic NPC patients (median follow-up time, 67.3 months) collected from the NPC-specific database in our institution; detailed information about the cohort and the database are presented in Additional file 1: Table S2 and Additional file 2: Supplementary Methods [38, 57–62]. The fitness of exponential, Gompertz, log-logistic, log-normal, and Weibull distributions were evaluated [63]. Specifically, the log-normal distribution was applied to the LR data, while the Gompertz distribution was applied to the RR and DM data, as they demonstrated the best fit based on the Akaike information criterion [63, 64]. The age-specific background mortality rate was generated from the China life table (Additional file 1: Table S3) [46]. The starting age of 45 in the base-case analysis was the median age of the 10,097 patients. We assumed that undetected early-stage LR, RR, and DM had monthly probabilities of 10%, 10%, and 20%, respectively, to progress to advanced-stage recurrence based on the values utilized in a published study [20]. Other probabilities and clinical utilities for different health states were derived from published studies and, in few cases, from expert opinion if published data were unavailable (Additional file 1: Table S1). The literature search and data extraction are detailed in Additional file 2: Supplementary Methods. All transition probabilities were transformed into monthly scales via the declining exponential approximation of life-expectancy equation: *μ* = − 1/*t* × ln(S) [65, 66].

### Model validation

To evaluate the ability of the Markov model to accurately describe the disease processes of NPC, we validated the model in two aspects. First, we compared the model-simulated disease recurrence patterns (LR, RR, and DM) against the real-world recurrence patterns in the NPC cohort of 10,097 patients. The model-simulated recurrence was generated from the Markov model using the base-case parameters, while the real-world recurrence was calculated using the Kaplan-Meier method. Second, we compared the model-predicted overall survival under the five surveillance strategies in this study with the real-world observed overall survival in the NPC cohort (Kaplan-Meier method). Although the patients in real-world clinical practice might be prescribed various follow-up modalities and intervals different from this study, the similarity between the model-predicted and observed survival could partially confirm the model’s ability to reproduce the natural history of NPC patients because the recurrence patterns in the model were supposed to be similar to those in the real-world cohort. To better mimic the real-world circumstance consisting of various follow-up methods, we calculated the model-predicted overall survival using the average of the overall survival of the five surveillance strategies in the model.

### Costs

Costs were derived from the 2019 Medical Insurance Administration Bureau of Guangzhou, China, or published studies from the societal perspective (Additional file 1: Table S1). Direct medical costs included costs of follow-up and salvage treatment. The costs assigned to a false-positive cfEBV DNA test or routine follow-up included the costs of additional imaging studies, while those assigned to a false-positive imaging study included the costs of nasopharyngoscopy- or CT-guided biopsy, pre-procedure labs, and pathology processing. Patients with early-stage LR would undergo endoscopic nasopharyngectomy (ENPG, 70%) or reirradiation (30%) [30], while patients with advanced-stage LR would undergo reirradiation. Patients with RR would undergo neck dissection, while patients with DM would receive salvage chemotherapy. The societal costs included costs of transportation, accommodations, meals, and wage loss related to patients’ and caregivers’ time off work [23, 53–55]. All costs were inflated to 2019 using the medical care component of the Consumer Price Index in China and were converted into 2019 US dollars (exchange rate, 1.00 USD = 6.91 CNY).

### Sensitivity analysis

One-way sensitivity analyses were applied to parameters over plausible ranges derived from published literature or a deviation of 20% from the base-case values (Additional file 1: Table S1) [53, 64]. Probabilistic sensitivity analyses (PSA) were conducted using 10,000 Monte Carlo simulations, where all parameters were varied simultaneously based on specific probability distributions (beta distribution for probabilities and utilities; gamma distribution for costs). Cost-effectiveness acceptability curves were constructed based on the strategies’ NHB generated from PSA. Additionally, two scenario analyses were performed by varying the surveillance arrangements: every 3 months in years 1–2 and every 6 months in years 3–5 according to the Radiation Therapy Oncology Group (RTOG) [67, 68]; every 2 months in year 1, every 4 months in year 2, and every 6 months in years 3–5 according to the NCCN guidelines for head and neck cancer (Version 1.2021) [19].

## Results

### Model validation

The Markov model-simulated LR-free survival, RR-free survival, and DM-free survival showed good agreement with the real-world disease recurrence patterns (Additional file 1: Fig. S2). In addition, the absolute differences between the model-predicted 1-year, 3-year, and 5-year overall survival and those of the real-world observation were within 2% in all disease stages, which indicated that the patient’s outcomes were comparable between the Markov model and the real-world observation (Additional file 1: Table S4). Collectively, these validation results demonstrated that the Markov model could accurately describe the disease processes of NPC.

### Base-case analysis

The results of the base-case analysis are summarized in Table 1 and Additional file 1: Table S5. With the assistance of cfEBV DNA, cfEBV DNA-guided imaging strategies were considerably more cost-effective than routine imaging strategies across all disease stages. Specifically, compared with cfEBV DNA-guided MRI + CT + BS, the ICERs of routine MRI + CT + BS or routine PET/CT were both exceeding the willingness-to-pay threshold of $100,000/QALY, ranging from $179,508/QALY in stage IV to $1,443,575/QALY in stage I (Additional file 1: Table S5). Similarly, the ICERs of routine imaging strategies compared with cfEBV DNA-guided PET/CT also went far beyond the willingness-to-pay threshold, ranging from $262,724/QALY in stage IV to $3,882,176/QALY in stage I, and the routine MRI + CT + BS was dominated (less effective but more costly than another strategy) by cfEBV DNA-guided PET/CT in stage IV (Additional file 1: Table S5). Moreover, the NHBs of cfEBV DNA-guided imaging strategies were also greater than those of routine imaging strategies (Table 1). For example, the NHBs of cfEBV DNA-guided MRI + CT + BS and PET/CT were 12.066/QALY and 12.061/QALY in stage II, respectively, larger than those of routine MRI + CT + BS (12.029/QALY) and PET/CT (11.979/QALY).

**Table 1.** Base-case cost-effectiveness analysis

	Total values		Incremental values^**b**^		ICER ($/QALY)	NHB^**c**^ (QALY)
Surveillance strategy^**a**^	Cost ($)	Effectiveness (QALY)	Costs ($)	Effectiveness (QALY)		
Stage I						
Routine clinical physical examination	5664	13.223	—	—	—	**13.166**
cfEBV DNA-guided MRI + CT + BS	6902	13.233	1239	0.010	119,368	13.164
cfEBV DNA-guided PET/CT	8113	13.236	1210	0.002	518,871	13.155
Routine MRI + CT + BS	11,546	13.237	3433	0.001	3,882,176	13.121
Routine PET/CT	17,458	13.241	5913	0.004	1378,484	13.066
Stage II						
Routine clinical physical examination	6694	12.121	—	—	—	12.054
cfEBV DNA-guided MRI + CT + BS	8341	12.150	1647	0.029	57,308	**12.066**
cfEBV DNA-guided PET/CT	9719	12.158	1378	0.009	162,041	12.061
Routine MRI + CT + BS	13,098	12.160	3379	0.002	1,923,004	12.029
Routine PET/CT	19,470	12.174	6373	0.014	454,793	11.979
Stage III						
Routine clinical physical examination	8421	11.452	—	—	—	11.367
cfEBV DNA-guided MRI + CT + BS	10,562	11.497	2141	0.046	46,860	**11.392**
cfEBV DNA-guided PET/CT	12,259	11.513	1697	0.016	108,549	11.390
Routine MRI + CT + BS	16,149	11.514	3891	0.001	2,780,611	11.353
Routine PET/CT	23,765	11.538	7615	0.024	318,452	11.300
Stage IV						
Routine clinical physical examination	10,101	9.869	—	—	—	9.768
cfEBV DNA-guided MRI + CT + BS	12,845	9.947	2744	0.079	34,906	9.819
cfEBV DNA-guided PET/CT	14,788	9.978	1944	0.031	62,269	**9.831**
Routine MRI + CT + BS	18,363	9.978	3574	-0.001	Dominated^d^	9.794
Routine PET/CT	26,342	10.022	11,553	0.044	262,724	9.759

Abbreviations: *BS*, bone scintigraphy; *cfEBV*, cell-free Epstein-Barr virus; *CT*, computed tomography; *ICER*, incremental cost-effectiveness ratio; *MRI*, magnetic resonance imaging; *NHB*, net health benefit; *PET/CT*, positron emission tomography/computed tomography; *QALY*, quality-adjusted life-year

^a^ Routine clinical physical examination consists of history and physical examinations, complete blood counts, comprehensive metabolic panels and nasopharyngoscopies. Other strategies also include routine clinical physical examination. Please see the manuscript for the full description of each strategy

^b^ Incremental values were compared with the previous less costly and nondominated strategy

^c^ Calculated at the willingness-to-pay threshold of $100,000 using the following formula: effectiveness − cost/willingness-to-pay. Strategies with the highest NHB values, highlighted in bold, are considered the most cost-effective

^d^ Refer to a strategy that is less effective and more costly than another strategy

**Fig. 1:**
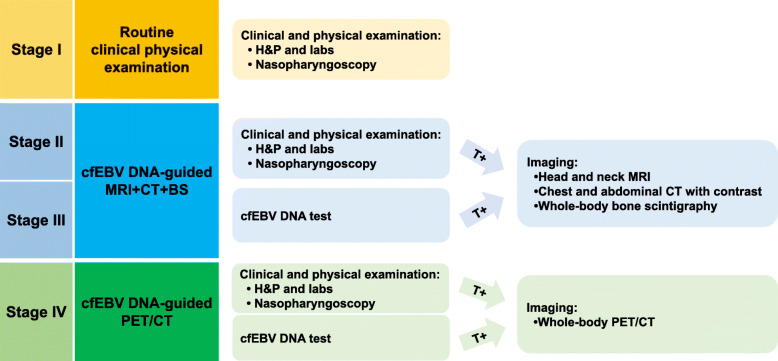
The cost-effective posttreatment surveillance strategy in each stage of nasopharyngeal carcinoma. The cfEBV DNA-guided MRI + CT + BS and cfEBV DNA-guided PET/CT refer to routine clinical and physical examinations and cfEBV DNA tests, followed by further imaging studies when the results are positive. Abbreviations: BS, bone scintigraphy; cfEBV, cell-free Epstein-Barr virus; CT, computed tomography; H&P, history and physical examination; MRI, magnetic resonance imaging; PET/CT, positron emission tomography/computed tomography; T, test

Next, we scrutinized the most cost-effective strategies in each stage of asymptomatic NPC patients (Table 1). For patients with stage I disease, cfEBV DNA-guided imaging strategies were only associated with a 0.01 gain in QALY compared with routine clinical physical examination. This translated to relatively large ICERs for cfEBV DNA-guided MRI + CT + BS ($119,368/QALY) and cfEBV DNA-guided PET/CT ($518,871/QALY), both surpassing the willingness-to-pay threshold. Therefore, routine follow-up was adequate for stage I NPC patients. However, in stage II and III, cfEBV DNA-guided MRI + CT + BS was the most cost-effective strategy, with ICERs of $57,308/QALY and $46,860/QALY, respectively, compared with routine clinical physical examination. Intriguingly, for patients with stage IV disease, cfEBV DNA-guided PET/CT became the most cost-effective, with an ICER of $62,269/QALY compared with cfEBV DNA-guided MRI + CT + BS. Likewise, the results of NHBs supported the same strategies mentioned above; the strategies with the highest NHBs were routine clinical physical examination in stage I (NHB, 13.166/QALY), cfEBV DNA-guided MRI + CT + BS in stage II (12.066/QALY) and stage III (11.392/QALY), and cfEBV DNA-guided PET/CT in stage IV (9.831/QALY). The preferred strategy in each stage is summarized in Fig. 1.

To further clarify the superiority of cfEBV DNA-guided imaging strategies over routine imaging strategies, we compared their average number of imaging studies required to detect one disease recurrence (Table 2). While routine imaging strategies resulted in a 5-year total of 97,493 to 118,328 imaging studies being performed per 10,000 patients, the cfEBV DNA-guided imaging strategies only required 19,676 to 25,893 imaging studies to detect a comparable number of recurrences as routine imaging strategies. Ultimately, compared with routine imaging strategies, cfEBV DNA-guided imaging strategies only required approximately one quarter of the imaging studies to detect one recurrence. For example, in stage II, routine MRI + CT + BS and routine PET/CT needed 121 and 106 imaging studies to detect one recurrence, respectively, while the guidance of cfEBV DNA decreased the number of imaging studies to 25 and 23, respectively. Therefore, cfEBV DNA-guided imaging strategies were the most favorable option for stage II to IV NPC patients. However, for stage I NPC patients, although the total imaging studies of cfEBV DNA-guided imaging strategies were remarkably fewer than routine imaging strategies, they still required a fair number of studies per recurrence detection (cfEBV DNA-guided MRI + CT + BS, 94; cfEBV DNA-guided PET/CT, 89) due to low recurrence probabilities, suggesting that, despite the guidance of cfEBV DNA, imaging was still unfavorable for them. This result was consistent with the cost-effectiveness analysis mentioned above that routine clinical physical examination was enough for patients with stage I disease.

**Table 2:** Number of disease recurrences detected by imaging studies in 5 years per 10,000 patients

Surveillance strategy	Imaging-detected local relapse	Imaging-detected regional relapse	Imaging-detected distant metastasis	Total imaging studies performed	Imaging studies per recurrence^**a**^	***P***^**b**^
Stage I						
cfEBV DNA-guided MRI + CT + BS	102	20	87	19,703	94	
cfEBV DNA-guided PET/CT	103	21	98	19,676	89	
Routine MRI + CT + BS	112	23	78	97,493	457	< .001
Routine PET/CT	119	24	99	97,519	405	< .001
Stage II						
cfEBV DNA-guided MRI + CT + BS	199	306	354	21,265	25	
cfEBV DNA-guided PET/CT	202	307	399	21,170	23	
Routine MRI + CT + BS	216	316	309	101,670	121	< .001
Routine PET/CT	228	319	410	101,595	106	< .001
Stage III						
cfEBV DNA-guided MRI + CT + BS	344	339	596	25,010	20	
cfEBV DNA-guided PET/CT	349	340	671	24,855	18	
Routine MRI + CT + BS	368	349	538	118,328	94	< .001
Routine PET/CT	389	350	672	118,204	84	< .001
Stage IV						
cfEBV DNA-guided MRI + CT + BS	571	484	1166	25,893	12	
cfEBV DNA-guided PET/CT	580	486	1324	25,598	11	
Routine MRI + CT + BS	610	502	1101	117,829	53	< .001
Routine PET/CT	640	508	1372	117,440	47	< .001

Abbreviations: *BS*, bone scintigraphy; *cfEBV*, cell-free Epstein-Barr virus; *CT*, computed tomography; *MRI*, magnetic resonance imaging; *PET/CT*, positron emission tomography/computed tomography

^a^ Indicate the number of imaging studies required to detect one recurrence, which was calculated by dividing the number of total imaging studies by the number of imaging-detected disease recurrences (including local relapse, regional relapse and distant metastasis).

^b^ Two-sided *P* values were calculated using Welch’s *t*-test for comparisons between cfEBV DNA-guided MRI + CT + BS and routine MRI + CT + BS; cfEBV DNA-guided PET/CT and routine PET/CT

### Sensitivity analysis

**Fig. 2:**
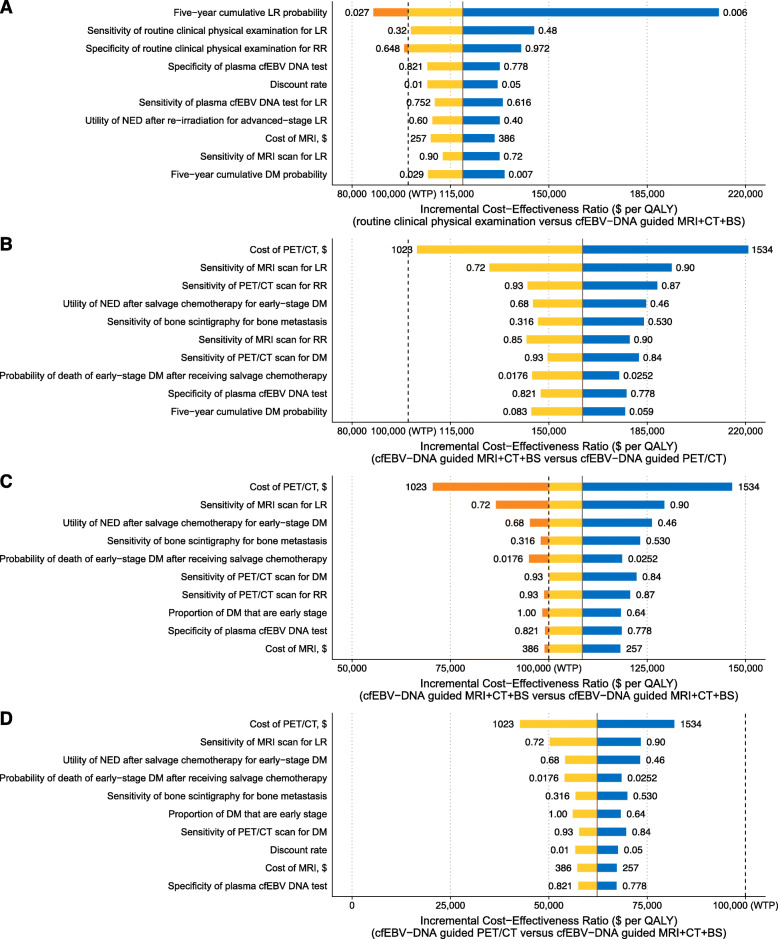
Tornado diagram of one-way sensitivity analysis. The figure depicts the influence of the variation of each parameter on the ICERs between **A** routine clinical physical examination and cfEBV DNA-guided MRI + CT + BS in patients with stage I NPC; cfEBV DNA-guided MRI + CT + BS and cfEBV DNA-guided PET/CT in patients with stage **B** II, **C** III, and **D** IV NPC. The parameters accounting for the top 10 uncertainties in each comparison are displayed. The blue bars and red bars illustrate the ICERs that are greater and less than the base-case ICERs, respectively. The orange bars indicate that the ICERs go across the willingness-to-pay threshold, leading to a switch of the most cost-effective strategy. The numbers on both sides of the bars indicate the range of each parameter used in the sensitivity analysis. The solid and dashed lines represent the ICERs in the base-case analysis and the willingness-to-pay threshold of $100,000 per quality-adjusted life-year, respectively. Abbreviations: BS, bone scintigraphy; cfEBV, cell-free Epstein-Barr virus; CT, computed tomography; DM, distant metastasis; ICER, incremental cost-effectiveness ratio; LR, local relapse; MRI, magnetic resonance imaging; NPC, nasopharyngeal carcinoma; PET/CT, positron emission tomography/computed tomography; RR, regional relapse; WTP, willingness-to-pay

To evaluate the results’ robustness, sensitivity analyses were performed on all probabilities, utilities, and costs. In one-way sensitivity analyses, cfEBV DNA-guided imaging strategies were consistently more cost-effective than routine imaging strategies across the range of all parameters (Additional file 1: Fig. S3–6). However, the optimal surveillance strategy in each stage was sensitive to several parameters when compared to the next more costly strategies, with key parameters displayed in Fig. 2A–D.

In stage I, the optimal strategy was sensitive to the LR probabilities and the specificity of routine clinical physical examination for RR (Fig. 2A). When increasing the LR probabilities to 2.1% or decreasing the specificity of routine clinical physical examination for RR to 65.9%, the preferred strategy would switch from routine clinical physical examination to cfEBV DNA-guided MRI + CT + BS. Nevertheless, all ICERs remained higher than $87,000/QALY when varying the parameters. In stage II–IV, the cost of PET/CT was the principal determinant of the ICERs (Fig. 2B–D). Specifically, if the PET/CT's cost was reduced to $1221, the cfEBV DNA-guided PET/CT would become the optimal strategy in stage III. Conversely, although the cost of PET/CT also had the greatest impact on the ICERs in stage II and IV, varying it did not change the optimal surveillance strategies in the base-case analysis.

**Fig. 3:**
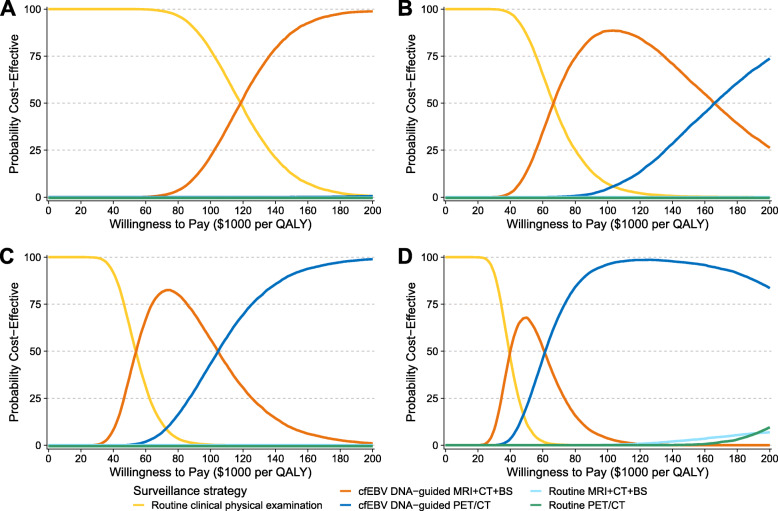
Cost-effectiveness acceptability curves. The figure illustrates the acceptability of each surveillance strategy in the probabilistic sensitivity analysis for stage I (**A**), II (**B**), III (**C**), and IV (**D**) NPC patients. The curves in different colors illustrate the probability of each surveillance strategy being the most cost-effective at a given willingness-to-pay threshold. Abbreviations: BS, bone scintigraphy; cfEBV, cell-free Epstein-Barr virus; CT, computed tomography; MRI, magnetic resonance imaging; NPC, nasopharyngeal carcinoma; PET/CT, positron emission tomography/computed tomography; QALY, quality-adjusted life-year

The PSA results are illustrated in the cost-effectiveness acceptability curves shown in Fig. 3A–D. With the willingness-to-pay threshold varying from 0 to $200,000/QALY, the probabilities of routine imaging strategies being the optimal strategies were almost zero in stage I–III and less than 10.0% in stage IV. At a willingness-to-pay threshold of $100,000/QALY, routine clinical physical examination was the most cost-effective in 78.2% of the simulations for stage I patients; cfEBV DNA-guided MRI + CT + BS was the most cost-effective in 88.4% and 56.3% of the simulations for stage II and III patients, respectively; and cfEBV DNA-guided PET/CT was the most cost-effective in 96.2% of the simulations for stage IV patients.

To examine the cost-effectiveness of cfEBV DNA-guided strategies under different follow-up arrangements, we performed two scenario analyses using the recommended arrangements from the RTOG and NCCN guidelines (Additional file 1: Table S6) [19, 67, 68]. For both scenarios, cfEBV DNA-guided imaging strategies were consistently more cost-effective than routine imaging strategies. The optimal surveillance strategy in each disease stage was in line with the base-case results.

## Discussion

To our knowledge, this is the first study to evaluate the liquid biopsy-based surveillance in NPC patients. We found that the cfEBV DNA-guided imaging strategies were remarkably more cost-effective than routine imaging strategies. Specifically, cfEBV DNA-guided MRI + CT + BS was cost-effective for stage II and III patients, while cfEBV DNA-guided PET/CT was cost-effective for stage IV patients. However, routine follow-up was sufficient for stage I NPC patients due to their low recurrence probabilities. Overall, our results highlight the feasibility of cfEBV DNA in the surveillance of NPC.

According to the current NCCN guideline, routine surveillance imaging was not recommended in asymptomatic patients [19], and our analysis agreed with it. However, directly halting imaging would result in delayed diagnoses of recurrence, especially for distant recurrence because many patients are asymptomatic [19, 69]. In fact, surveillance imaging was the most widely used follow-up modality in NPC, and 79% of clinicians would order routine imaging in clinical practice for reassurance, patient requests, etc. [70]. Consequently, undifferentiated imaging surveillance has resulted in considerable economic burdens and resource consumption. Given these circumstances, it is urgent to establish a convenient and reliable method that can distinguish patients at high risk of developing recurrence so as to focus imaging surveillance on them.

cfEBV DNA is an ideal biomarker with low expense and high sensitivity in suggesting disease recurrence. The ESMO and NCCN guidelines both recommended cfEBV DNA tests in NPC follow-up [17, 19]. Therefore, cfEBV DNA-guided imaging strategies are particularly promising. Using cfEBV DNA, patients at risk of recurrence can be identified in order to concentrate surveillance imaging on those who will benefit, thus sparing the unnecessary expense and radiation exposure for those who will not. Compared with routine imaging surveillance, targeting imaging to patients with positive cfEBV DNA gained similar survival benefits but only required one quarter of the imaging to detect one recurrence. This liquid biopsy-based surveillance strategy underscores the practice of precision medicine in the follow-up of cancer survivors, which has been investigated in several malignancies [71–73]. For example, Roschewski et al. [72] found that surveillance circulating tumor DNA could accurately suggest recurrence in diffuse large B cell lymphoma, leading to reduced disease burden at recurrence.

Until now, few studies have investigated the optimal follow-up modalities for each stage of NPC [30]. Consequently, despite the substantial heterogeneity of recurrence risk in different disease stages of NPC [1, 22], the ESMO and NCCN guideline failed to recommend stage-specific follow-up strategies [17, 19]. Zhou et al. [30] evaluated the cost-effectiveness of surveillance MRI and recommended annual MRI for patients with advanced tumor stage and no MRI for early stage. However, they did not consider distant recurrence, the predominant failure pattern in NPC [1]. Besides, they did not investigate cfEBV DNA and PET/CT, which have been widely adopted in clinical practice. Our study addressed these unmet needs. We found that cfEBV DNA-guided imaging surveillance is particularly preferable for stage II–IV NPC patients, where selected MRI + CT + BS or PET/CT were performed depending on their disease stages. However, for stage I patients, cfEBV DNA-guided imaging is not recommended due to their fairly good survival, with 10-year overall survival and recurrence-free survival both greater than 95% [74]. Hence, routine follow-up would be sufficient for stage I NPC patients.

Evidence has indicated that PET/CT was more effective than other imaging modalities in detecting recurrent NPC, especially for distant recurrence [19, 31, 34, 37, 75]. However, lacking guidance regarding when to perform PET/CT during NPC surveillance, clinicians have to be critically cautious about prescribing costly PET/CT when following up patients with relatively poor economic conditions. In this study, we found that cfEBV DNA could be a precise indicator to perform PET/CT for patients at risk of recurrence, which was cost-effective in stage IV NPC patients. In addition, we observed that the cost of PET/CT predominantly determined the ICERs between cfEBV DNA-guided PET/CT and cfEBV DNA-guided MRI + CT + BS in the sensitivity analyses. With the discounting of PET/CT, cfEBV DNA-guide PET/CT will be increasingly cost-effective. Therefore, the tradeoffs between the two strategies should be continuously recalibrated to reflect the updated cost of PET/CT in clinical practice, especially among stage III NPC patients where cfEBV DNA-guided PET/CT was near the most cost-effective strategy.

With the growing recognition that cfEBV DNA possessed favorable performance in detecting tumor recurrence [13–16, 76], we proposed cfEBV DNA-guided surveillance strategies as references for patients and clinicians in NPC follow-up. Nevertheless, our recommendations should be implemented cautiously. First, although cfEBV DNA demonstrates high sensitivity in detecting RR and DM, it is relatively insensitive to LR [13, 14, 16, 76]. Therefore, cfEBV DNA results should be interpreted with the history and physical examinations and nasopharyngoscopies to comprehensively evaluate the local conditions [76]. Second, cfEBV DNA levels might elevate even before recurrences can be detected on imaging [16, 76]. Hence, patients with detectable cfEBV DNA but negative imaging might require subsequent cfEBV DNA tests (e.g., another cfEBV DNA test 1–3 months later) to distinguish much earlier-stage recurrence from false-positive results, especially for those with high recurrence risk [16].

Several limitations need to be noted. First, the inherent shortcoming of the decision-analytic model was the model inputs determined from various sources. We therefore conducted extensive sensitivity analyses to clarify the uncertainty, and the cfEBV DNA-guided imaging strategy was confirmed to be robustly cost-effective in the sensitivity analysis. Second, the model did not account for secondary recurrences because our study population was patients achieving CR to the primary treatment for NPC. Patients who have experienced recurrence will manifest distinct risks of disease failure, and thus may require different surveillance strategies [8, 77]. Third, since the study is from the Chinese societal perspective, the results might vary with different medical and societal costs. Accordingly, similar analyses in other regions are warranted. Last but not least, the study does not account for non-endemic NPC. NPC in endemic areas, including southern China, Southeast Asia, and North and East Africa, constitutes more than 80% of new cases worldwide [78–80], which are mostly the non-keratinising subtype (> 95%) and are invariably associated with EBV infection [81]. However, the keratinising squamous subtype is more common in regions where NPC is non-endemic, e.g., North America and northern Europe [81, 82]. Evidence has indicated that patients with the keratinising squamous subtype tend to have worse survival, poorer local control, and less distant failures than those with the non-keratinising subtype; and their association with EBV infection is relatively low [81–84]. Therefore, the applicability of cfEBV DNA-guided surveillance strategies should be carefully re-evaluated in non-endemic areas.

## Conclusions

In this model-based analysis of follow-up strategies in NPC, we proposed cfEBV DNA-guided imaging strategies that were the most cost-effective for stage II–IV patients. The liquid biopsy-based surveillance strategies are of high clinical feasibility, which simultaneously takes patient outcomes and resource consumption into account. In the precision medicine era, these results would add new insight into the momentum of liquid biopsy in the surveillance of cancer survivors.

## Supplementary Information

**Additional file 1: Table S1.** Model parameters. **Table S2.** Characteristics of 10,097 patients with nonmetastatic nasopharyngeal carcinoma. **Table S3.** Age-specific background mortality rate. **Table S4.** Model validation of the Markov model-predicted overall survival compared with the real-world observed overall survival. **Table S5.** Base-case cost-effectiveness analysis comparing cfEBV DNA-guided imaging strategies with routine imaging strategies. **Table S6.** Cost-effectiveness scenario analyses using the RTOG and NCCN surveillance arrangements. **Fig. S1.** Schematic diagram of the surveillance strategies and the structure of the Markov model. **Fig. S2.** Validation of the Markov model. **Fig. S3.** Tornado diagram of one-way sensitivity analysis for stage I NPC patients. **Fig. S4.** Tornado diagram of one-way sensitivity analysis for stage II NPC patients. **Fig. S5.** Tornado diagram of one-way sensitivity analysis for stage III NPC patients. **Fig. S6.** Tornado diagram of one-way sensitivity analysis for stage IV NPC patients.

**Additional file 2.** Supplementary methods.

## Data Availability

The patient-level data utilized in the study, including patient characteristics, treatments, and survival outcomes, have been deposited in the Research Data Deposit public platform with the accession RDD number: RDDA2021001822 (https://www.researchdata.org.cn/Search.aspx?Num = RDDA2021001822); and the data are available from the corresponding authors on reasonable request. All the other data of the study are available within the article and the additional files and from the corresponding authors upon reasonable request.

## Abbreviations

BS: Bone scintigraphy
cfEBV: Circulating cell-free Epstein-Barr virus
ESMO: European Society for Medical Oncology
ICER: Incremental cost-effectiveness ratio
NPC: Nasopharyngeal carcinoma
NCCN: National Comprehensive Cancer Network
NHB: Net health benefit
PET/CT: Positron emission tomography/computed tomography
PSA: Probabilistic sensitivity analyses
QALY: Quality-adjusted life-year
