# Supplementary material for: Liquid biopsy posttreatment surveillance in endemic nasopharyngeal carcinoma: a cost-effective strategy to integrate circulating cell-free Epstein-Barr virus DNA
Source: BMC Med. 2021 Aug 26;19:193. doi: 10.1186/s12916-021-02076-4 (PMC8390246; doi:10.1186/s12916-021-02076-4)
Supplement: Supplementary file 1 — Additional file 1: Table S1. Model parameters. Table S2. Characteristics of 10,097 patients with nonmetastatic nasopharyngeal carcinoma. Table S3. Age-specific background mortality rate. Table S4. Model validation of the Markov model-predicted overall survival compared with the real-world observed overall survival. Table S5. Base-case cost-effectiveness analysis comparing cfEBV DNA-guided imaging strategies with routine imaging strategies. Table S6. Cost-effectiveness scenario analyses using the RTOG and NCCN surveillance arrangements. Fig. S1. Schematic diagram of the surveillance strategies and the structure of the Markov model. Fig. S2. Validation of the Markov model. Fig. S3. Tornado diagram of one-way sensitivity analysis for stage I NPC patients. Fig. S4. Tornado diagram of one-way sensitivity analysis for stage II NPC patients. Fig. S5. Tornado diagram of one-way sensitivity analysis for stage III NPC patients. Fig. S6. Tornado diagram of one-way sensitivity analysis for stage IV NPC patients. [file 12916_2021_2076_MOESM1_ESM.docx]

**Additional File 1:**

**Liquid Biopsy Posttreatment Surveillance in Endemic Nasopharyngeal Carcinoma: A Cost-Effective Strategy to Integrate Circulating Cell-Free Epstein-Barr Virus DNA**

**Additional file 1: Tables S1–S6**

**Table S1. Model Parameters**

| **Parameter** | **Base-Case Estimate** | **Range** | **Distribution** | **Reference** |
| --- | --- | --- | --- | --- |
| Test performance |  |  |  |  |
| Detection of local relapse |  |  |  |  |
| Sensitivity of routine clinical physical examination^†^ | 40.0% | 32.0%–48.0% | Beta | [28-30] |
| Specificity of routine clinical physical examination^†^ | 90.0% | 85.8%–95.0% | Beta | [28-30] |
| Sensitivity of plasma cfEBV DNA test | 68.8% | 61.6%–75.2% | Beta | [16] |
| Specificity of plasma cfEBV DNA test | 80.0% | 77.8%–82.1% | Beta | [16] |
| Sensitivity of MRI scan with contrast (head and neck) | 83.0% | 72.0%–90.0% | Beta | [31] |
| Specificity of MRI scan with contrast (head and neck) | 78.0% | 70.0%–85.0% | Beta | [31] |
| Sensitivity of PET/CT scan | 92.0% | 89.0%–95.0% | Beta | [31] |
| Specificity of PET/CT scan | 89.0% | 84.0%–93.0% | Beta | [31] |
| Detection of regional relapse |  |  |  |  |
| Sensitivity of routine clinical physical examination^†^ | 74.0% | 59.2%–88.8% | Beta | [32] |
| Specificity of routine clinical physical examination^†^ | 81.0% | 64.8%–97.2% | Beta | [32] |
| Sensitivity of plasma cfEBV DNA test | 80.2% | 73.3%–85.7% | Beta | [16] |
| Specificity of plasma cfEBV DNA test | 80.0% | 77.8%–82.1% | Beta | [16] |
| Sensitivity of MRI scan with contrast (head and neck) | 88.0% | 85.0%–90.0% | Beta | [33] |
| Specificity of MRI scan with contrast (head and neck) | 95.0% | 93.0%–97.0% | Beta | [33] |
| Sensitivity of PET/CT scan | 90.0% | 87.0%–93.0% | Beta | [34] |
| Specificity of PET/CT scan | 92.0% | 89.0%–95.0% | Beta | [34] |
| Detection of distant metastasis |  |  |  |  |
| Sensitivity of plasma cfEBV DNA test | 91.1% | 87.6%–93.7% | Beta | [16] |
| Specificity of plasma cfEBV DNA test | 80.0% | 77.8%–82.1% | Beta | [16] |
| Sensitivity of CT scan with contrast (chest and abdomen) | 80.0% | 73.0%–85.0% | Beta | [35] |
| Specificity of CT scan with contrast (chest and abdomen) | 94.0% | 89.0%–97.0% | Beta | [35] |
| Sensitivity of bone scintigraphy | 42.0% | 31.6%–53.0% | Beta | [36, 37] |
| Specificity of bone scintigraphy | 98.8% | 97.9%–99.4% | Beta | [36, 37] |
| Sensitivity of PET/CT scan | 89.0% | 84.0%–93.0% | Beta | [34] |
| Specificity of PET/CT scan | 97.0% | 96.0%–98.0% | Beta | [34] |
| Transition probabilities |  |  |  |  |
| Local relapse |  |  |  | NPC-specific database [38] |
| Stage I | 1.7% by 5 years | 0.6%–2.7% | Beta |  |
| Log-normal model parameters | μ=7.850, σ=1.773 |  |  |  |
| Stage II | 3.3% by 5 years | 2.7%–4.4% | Beta |  |
| Log-normal model parameters | μ=8.262, σ=2.274 |  |  |  |
| Stage III | 5.7% by 5 years | 5.2%–6.6% | Beta |  |
| Log-normal model parameters | μ=7.258, σ=1.997 |  |  |  |
| Stage IV | 10.2% by 5 years | 9.5%–11.8% | Beta |  |
| Log-normal model parameters | μ=6.523, σ=1.912 |  |  |  |
| Regional relapse |  |  |  | NPC-specific database [38] |
| Stage I | 0.4% by 5 years | 0.0%–0.9% | Beta |  |
| Gompertz model parameters | λ=0.0001, γ=0.0385 |  |  |  |
| Stage II | 4.3% by 5 years | 3.5%–5.5% | Beta |  |
| Gompertz model parameters | λ=0.0012, γ=-0.0190 |  |  |  |
| Stage III | 4.9% by 5 years | 4.5%–5.8% | Beta |  |
| Gompertz model parameters | λ=0.0012, γ=-0.0118 |  |  |  |
| Stage IV | 7.2% by 5 years | 6.6%–8.5% | Beta |  |
| Gompertz model parameters | λ=0.0019, γ=-0.0158 |  |  |  |
| Distant metastasis |  |  |  | NPC-specific database [38] |
| Stage I | 1.7% by 5 years | 0.7%–2.9% | Beta |  |
| Gompertz model parameters | λ=0.0007, γ=-0.0322 |  |  |  |
| Stage II | 6.8% by 5 years | 5.9%–8.3% | Beta |  |
| Gompertz model parameters | λ=0.0023, γ=-0.0246 |  |  |  |
| Stage III | 10.7% by 5 years | 9.8%–11.6% | Beta |  |
| Gompertz model parameters | λ=0.0037, γ=-0.0251 |  |  |  |
| Stage IV | 20.6% by 5 years | 19.4%–22.3% | Beta |  |
| Gompertz model parameters | λ=0.0078, γ=-0.0273 |  |  |  |
| Transition probabilities (monthly) |  |  |  |  |
| Proportion of disease recurrence that are early stage |  |  |  |  |
| For local relapse | 0.90 | 0.72–1.00 | Beta | [20] |
| For regional relapse | 0.90 | 0.72–1.00 | Beta | [20] |
| For distant metastasis | 0.80 | 0.64–1.00 | Beta | [20] |
| Probability of progression from early to advanced-stage recurrence  if the recurrence is undetected in one cycle |  |  |  |  |
| For local relapse | 0.10 | 0.08–0.12 | Beta | [20] |
| For regional relapse | 0.10 | 0.08–0.12 | Beta | [20] |
| For distant metastasis | 0.20 | 0.16–0.24 | Beta | [20] |
| Proportion of receiving ENPG at early-stage local relapse | 0.70 | 0.56–0.84 | Beta | [30] |
| Probability of death if persistent disease |  |  |  |  |
| Early-stage local relapse | 0.0489  (70.0% by 2 years) | 0.0391–0.0587 | Beta | [20] |
| Advanced-stage local relapse | 0.0915  (90.0% by 2 years) | 0.0732–0.1098 | Beta | [20] |
| Early-stage regional relapse | 0.0489  (70.0% by 2 years) | 0.0391–0.0587 | Beta | [20] |
| Advanced-stage regional relapse | 0.0915  (90.0% by 2 years) | 0.0732–0.1098 | Beta | [20] |
| Early-stage distant metastasis | 0.1173  (95.0% by 2 years) | 0.0939–0.1408 | Beta | Assumption |
| Advanced-stage distant metastasis | 0.2209  (95.0% by 1 years) | 0.1767–0.2651 | Beta | Assumption |
| Probability of death of disease recurrence after treatment |  |  |  |  |
| Early-stage local relapse  after receiving ENPG | 0.0052  (27.0% by 5 years) | 0.0021–0.0096 | Beta | [39] |
| Early-stage local relapse  after receiving reirradiation | 0.0060  (30.4% by 5 years) | 0.0033–0.0071 | Beta | [4, 40, 41] |
| Advanced-stage local relapse  after receiving reirradiation | 0.0176  (65.5% by 5 years) | 0.0160–0.0213 | Beta | [40-42] |
| Early-stage regional relapse  after receiving neck dissection | 0.0048  (25.1% by 5 years) | 0.0025–0.0070 | Beta | [43, 44] |
| Advanced-stage regional relapse  after receiving neck dissection | 0.0155  (60.9% by 5 years) | 0.0117–0.0273 | Beta | [43, 44] |
| Early-stage distant metastasis  after receiving salvage chemotherapy | 0.0218  (73.4% by 5 years) | 0.0176–0.0252 | Beta | [3, 45] |
| Advanced-stage distant metastasis  after receiving salvage chemotherapy | 0.0458  (81.5% by 3 years) | 0.0422–0.0516 | Beta | [3, 45] |
| Background mortality rate | Age specific |  |  | [46] |
| Base-case age | 45 | 36­–54 | Gamma | NPC-specific database [38] |
| Discount rate (annual) | 0.03 | 0.01–0.05 | Beta | [23] |
| Health-state utilities (annual), QALY |  |  |  |  |
| NED after primary treatment | 0.76 | 0.62–0.90 | Beta | [30, 47, 48] |
| Local relapse | 0.35 | 0.28–0.42 | Beta | [20] |
| Regional relapse | 0.40 | 0.32–0.48 | Beta | [20] |
| Distant metastasis | 0.30 | 0.24–0.36 | Beta | [20, 49, 50] |
| NED after ENPG for early-stage local relapse | 0.60 | 0.48–0.72 | Beta | [30] |
| NED after reirradiation for early-stage local relapse | 0.55 | 0.44–0.66 | Beta | [30] |
| NED after reirradiation for advanced-stage local relapse | 0.50 | 0.40–0.60 | Beta | [30] |
| NED after neck dissection for early-stage local relapse | 0.70 | 0.56–0.84 | Beta | [20, 30] |
| NED after neck dissection for advanced-stage local relapse | 0.60 | 0.48–0.72 | Beta | [20, 30] |
| NED after salvage chemotherapy  for early-stage distant metastasis | 0.57 | 0.46–0.68 | Beta | [50, 51] |
| NED after salvage chemotherapy  for advanced-stage distant metastasis | 0.47 | 0.38–0.56 | Beta | [50, 51] |
| Costs ($) |  |  |  |  |
| Routine follow-up clinic visit | 68.2 | 55–82 | Gamma | MIAB |
| Complete blood count | 2.7 |  |  |  |
| Comprehensive metabolic panel | 37.5 |  |  |  |
| Nasopharyngoscopy | 28.0 |  |  |  |
| Plasma cfEBV DNA test | 21.3 | 17–26 | Gamma | MIAB |
| MRI scan with contrast (head and neck) | 321.7 | 257–386 | Gamma | MIAB |
| CT scan with contrast | 177.0 | 142–212 | Gamma | MIAB |
| CT chest | 88.5 |  |  |  |
| CT abdomen | 88.5 |  |  |  |
| Bone scintigraphy | 121.3 | 97–146 | Gamma | MIAB |
| PET/CT scan | 1278.4 | 1023–1534 | Gamma | MIAB |
| Work-up for imaging detected local relapse | 146.0 | 117–175 | Gamma | MIAB |
| Pre-procedure labs | 74.1 |  |  |  |
| Nasopharyngoscopy-guided biopsy | 43.0 |  |  |  |
| Pathology processing | 28.9 |  |  |  |
| Work-up for imaging detected regional relapse | 146.8 | 117–176 | Gamma | MIAB |
| Pre-procedure labs | 74.1 |  |  |  |
| CT-guided biopsy | 43.8 |  |  |  |
| Pathology processing | 28.9 |  |  |  |
| Work-up for imaging detected distant metastasis | 159.2 | 127–191 | Gamma | MIAB |
| Pre-procedure labs | 74.1 |  |  |  |
| CT-guided biopsy | 56.2 |  |  |  |
| Pathology processing | 28.9 |  |  |  |
| Endoscopic nasopharyngectomy for early-stage local relapse | 3310.4 | 2648–3972 | Gamma | [30, 52] |
| Reirradiation for early-stage local relapse | 19486.8 | 15589–23384 | Gamma | [30, 52] |
| Reirradiation for advanced-stage local relapse | 27988.0 | 22390–33586 | Gamma | [30] |
| Neck dissection for regional relapse | 5114.7 | 4092–6138 | Gamma | [30] |
| Salvage chemotherapy for distant metastasis | 28986.0 | 23189–34783 | Gamma | [20] |
| Societal costs related to each follow-up visit | 475.8 | 147–964 | Gamma | [23, 53-56] |
| Direct non-health care cost^‡^ | 228.9 |  |  |  |
| Indirect cost^§^ | 246.9 |  |  |  |

Note. All costs were inflated to year 2019 according to the medical care component of the Consumer Price Index in China and were converted into 2019 US dollars.

Abbreviations: cfEBV, cell-free Epstein-Barr virus; CR, complete remission; CT, computed tomography; ENPG, endoscopic nasopharyngectomy; MRI, magnetic resonance imaging; MIAB, Medical Insurance Administration Bureau of Guangzhou, China; NED, no evidence of disease; PET/CT, positron emission tomography/computed tomography; QALY, quality-adjusted life-year.

^†^ Routine clinical physical examination includes history and physical examinations, complete blood counts, comprehensive metabolic panels and surveillance nasopharyngoscopies.

^‡^ Includes the costs of transportation, accommodations and meals.

^§^ Refers to wage loss related to patients' and caregivers' time off work, which was calculated based on the China Statistical Yearbook 2019 [56] with the following formula [53, 55]: time off work * labor costs * mean participants.

**Table S2: Characteristics of 10,097 Patients with Nonmetastatic Nasopharyngeal Carcinoma**

| **Characteristics** | **No. of Patients (%)**  **(*N* = 10,097)** |
| --- | --- |
| Age, years |  |
| ≤45 | 5,305 (52.5) |
| >45 | 4,792 (47.5) |
| Sex |  |
| Male | 7,413 (73.4) |
| Female | 2,684 (26.6) |
| Histology, WHO type^†^ |  |
| I | 65 (0.6) |
| II | 205 (2.0) |
| III | 9,827 (97.3) |
| Smoking |  |
| No | 6,564 (65.0) |
| Yes | 3,533 (35.0) |
| Alcohol |  |
| No | 8,708 (86.2) |
| Yes | 1,389 (13.8) |
| Family history of NPC |  |
| No | 7,425 (73.5) |
| Yes | 2,672 (26.5) |
| Clinical stage^‡^ |  |
| I | 558 (5.5) |
| II | 1,787 (17.7) |
| III | 4,662 (46.2) |
| IV | 3,090 (30.6) |
| T stage^‡^ |  |
| T1 | 1,674 (16.6) |
| T2 | 1,629 (16.1) |
| T3 | 4,692 (46.5) |
| T4 | 2,102 (20.8) |
| N stage^‡^ |  |
| N0 | 1,582 (15.7) |
| N1 | 5,127 (50.8) |
| N2 | 2,158 (21.4) |
| N3 | 1,230 (12.2) |
| Treatment^§^ |  |
| RT alone | 1,234 (12.2) |
| IC+RT | 994 (9.8) |
| CCRT | 3,815 (37.8) |
| IC+CCRT | 4,054 (40.2) |

Abbreviations: CCRT, concurrent chemoradiotherapy; IC+CCRT, induction chemotherapy plus concurrent chemoradiotherapy; IC+RT, induction chemotherapy plus radiotherapy; N, node; NOS, not otherwise specified; NPC, nasopharyngeal carcinoma; RT, radiotherapy; T, tumor; WHO, World Health Organization.

^†^ WHO Type I refers to the keratinizing squamous cell carcinoma; WHO Type II refers to the differentiated non-keratinizing carcinoma; WHO Type III refers to the undifferentiated non-keratinizing carcinoma.

^‡^ According to the 8th edition of AJCC/UICC Staging System.

^§^ All patients received intensity-modulated radiotherapy.

**Table S3. Age-specific Background Mortality Rate**

| **Age Group** | **Background Yearly**  **Mortality Rate of Male** | **Background Yearly**  **Mortality Rate of Female** |
| --- | --- | --- |
| <1 year | 0.008 | 0.008 |
| 1–4 years | 0.000 | 0.000 |
| 5–9 years | 0.000 | 0.000 |
| 10–14 years | 0.000 | 0.000 |
| 15–19 years | 0.000 | 0.000 |
| 20–24 years | 0.001 | 0.000 |
| 25–29 years | 0.001 | 0.001 |
| 30–34 years | 0.001 | 0.001 |
| 35–39 years | 0.001 | 0.001 |
| 40–44 years | 0.002 | 0.001 |
| 45–49 years | 0.002 | 0.002 |
| 50–54 years | 0.004 | 0.003 |
| 55–59 years | 0.007 | 0.005 |
| 60–64 years | 0.014 | 0.010 |
| 65–69 years | 0.025 | 0.018 |
| 70–74 years | 0.045 | 0.032 |
| 75–79 years | 0.076 | 0.055 |
| 80–84 years | 0.111 | 0.092 |
| 85+ years | 0.200 | 0.180 |

Note. Age–specific background mortality rate were generated from the China life table [46].

**Table S4. Model Validation of the Markov Model-Predicted Overall Survival Compared with the Real-world Observed Overall Survival**

| **Outcome** | **Markov Model Prediction (%)^†^** | **Real-world Observation (%)^‡^** | **Absolute Difference (%)^§^** |
| --- | --- | --- | --- |
| 1-year overall survival |  |  |  |
| Stage I | 99.7 | 100.0 | 0.3 |
| Stage II | 98.6 | 99.7 | 1.1 |
| Stage III | 98.6 | 99.3 | 0.8 |
| Stage IV | 97.2 | 98.5 | 1.3 |
| 3-year overall survival |  |  |  |
| Stage I | 98.8 | 99.5 | 0.7 |
| Stage II | 95.0 | 96.5 | 1.6 |
| Stage III | 93.3 | 93.5 | 0.2 |
| Stage IV | 87.5 | 85.9 | 1.7 |
| 5-year overall survival |  |  |  |
| Stage I | 97.7 | 98.9 | 1.2 |
| Stage II | 91.1 | 92.9 | 1.7 |
| Stage III | 87.1 | 88.3 | 1.2 |
| Stage IV | 77.4 | 76.6 | 0.8 |

^†^ The Markov model-predicted overall survival was the average of the overall survival of the five surveillance strategies in this study.

^‡^ The real-world observed overall survival was calculated by the Kaplan-Meier method using the time-to-event data of the NPC cohort containing 10,097 nonmetastatic nasopharyngeal carcinoma patients.

^§^ The absolute difference was the absolute value of the "Markov Model Prediction" minus the "Real-world Observation".

**Table S5. Base-case Cost-effectiveness Analysis Comparing cfEBV DNA-guided Imaging Strategies with Routine Imaging Strategies**

|  | **Total Values** | |  | **Values Compared with**  **cfEBV DNA-guided MRI+CT+BS** | | |  | **Values Compared with**  **cfEBV DNA-guided PET/CT** | | |
| --- | --- | --- | --- | --- | --- | --- | --- | --- | --- | --- |
| **Surveillance Strategy^†^** | **Cost ($)** | **Effectiveness (QALY)** |  | **Costs ($)** | **Effectiveness (QALY)** | **ICER ($/QALY)** |  | **Costs ($)** | **Effectiveness (QALY)** | **ICER ($/QALY)** |
| Stage I |  |  |  |  |  |  |  |  |  |  |
| cfEBV DNA-guided MRI+CT+BS | 6,902 | 13.233 |  | — | — | — |  | — | — | — |
| cfEBV DNA-guided PET/CT | 8,113 | 13.236 |  | — | — | — |  | — | — | — |
| Routine MRI+CT+BS | 11,546 | 13.237 |  | 4,643 | 0.003 | 1,443,575 |  | 3,433 | 0.001 | 3,882,176 |
| Routine PET/CT | 17,458 | 13.241 |  | 10,556 | 0.008 | 1,406,378 |  | 9,346 | 0.005 | 1,806,438 |
| Stage II |  |  |  |  |  |  |  |  |  |  |
| cfEBV DNA-guided MRI+CT+BS | 8,341 | 12.150 |  | — | — | — |  | — | — | — |
| cfEBV DNA-guided PET/CT | 9,719 | 12.158 |  | — | — | — |  | — | — | — |
| Routine MRI+CT+BS | 13,098 | 12.160 |  | 4,757 | 0.010 | 463,550 |  | 3,379 | 0.002 | 1,923,004 |
| Routine PET/CT | 19,470 | 12.174 |  | 11,129 | 0.024 | 458,495 |  | 9,751 | 0.016 | 618,385 |
| Stage III |  |  |  |  |  |  |  |  |  |  |
| cfEBV DNA-guided MRI+CT+BS | 10,562 | 11.497 |  | — | — | — |  | — | — | — |
| cfEBV DNA-guided PET/CT | 12,259 | 11.513 |  | — | — | — |  | — | — | — |
| Routine MRI+CT+BS | 16,149 | 11.514 |  | 5,587 | 0.017 | 328,076 |  | 3,891 | 0.001 | 2,780,611 |
| Routine PET/CT | 23,765 | 11.538 |  | 13,202 | 0.041 | 322,455 |  | 11,506 | 0.025 | 454,551 |
| Stage IV |  |  |  |  |  |  |  |  |  |  |
| cfEBV DNA-guided MRI+CT+BS | 12,845 | 9.947 |  | — | — | — |  | — | — | — |
| cfEBV DNA-guided PET/CT | 14,788 | 9.978 |  | — | — | — |  | — | — | — |
| Routine MRI+CT+BS | 18,363 | 9.978 |  | 5,518 | 0.031 | 180,326 |  | 3,574 | -0.001 | Dominated^†^ |
| Routine PET/CT | 26,342 | 10.022 |  | 13,497 | 0.075 | 179,508 |  | 11,553 | 0.044 | 262,724 |

Abbreviations: BS, bone scintigraphy; cfEBV, cell-free Epstein-Barr virus; CT, computed tomography; ICER, incremental cost-effectiveness ratio; MRI, magnetic resonance imaging; PET/CT, positron emission tomography/computed tomography; QALY, quality-adjusted life-year.

^†^ Refer to a strategy that is less effective and more costly than another strategy.

**Table S6. Cost-effectiveness Scenario Analyses Using the RTOG and NCCN Surveillance Arrangements**

|  | **Total Values** | |  | **Incremental Values^‡^** | | **ICER**  **($/QALY)** | **NHB^§^**  **(QALY)** |
| --- | --- | --- | --- | --- | --- | --- | --- |
| **Surveillance Strategy^†^** | **Cost ($)** | **Effectiveness (QALY)** |  | **Costs ($)** | **Effectiveness (QALY)** |  |  |
| **RTOG Surveillance Protocol** |  |  |  |  |  |  |  |
| Stage I |  |  |  |  |  |  |  |
| Routine clinical physical examination | 7,930 | 13.228 |  | — | — | — | **13.149** |
| cfEBV DNA-guided MRI+CT+BS | 9,605 | 13.240 |  | 1,675 | 0.011 | 148,581 | 13.144 |
| cfEBV DNA-guided PET/CT | 11,302 | 13.243 |  | 1,697 | 0.003 | 559,745 | 13.130 |
| Routine MRI+CT+BS | 16,167 | 13.244 |  | 4,865 | 0.001 | 5,395,092 | 13.082 |
| Routine PET/CT | 24,478 | 13.249 |  | 8,311 | 0.005 | 1,693,937 | 13.004 |
| Stage II |  |  |  |  |  |  |  |
| Routine clinical physical examination | 8,441 | 12.143 |  | — | — | — | 12.058 |
| cfEBV DNA-guided MRI+CT+BS | 10,424 | 12.174 |  | 1,983 | 0.031 | 63,857 | **12.069** |
| cfEBV DNA-guided PET/CT | 12,168 | 12.184 |  | 1,743 | 0.011 | 165,896 | 12.063 |
| Routine MRI+CT+BS | 16,560 | 12.186 |  | 4,392 | 0.002 | 1,999,437 | 12.021 |
| Routine PET/CT | 24,672 | 12.202 |  | 8,113 | 0.016 | 506,109 | 11.956 |
| Stage III |  |  |  |  |  |  |  |
| Routine clinical physical examination | 8,903 | 11.462 |  | — | — | — | 11.373 |
| cfEBV DNA-guided MRI+CT+BS | 11,167 | 11.509 |  | 2,264 | 0.048 | 47,545 | **11.398** |
| cfEBV DNA-guided PET/CT | 12,957 | 11.525 |  | 1,790 | 0.016 | 111,362 | 11.396 |
| Routine MRI+CT+BS | 17,066 | 11.527 |  | 4,109 | 0.001 | 3,157,547 | 11.356 |
| Routine PET/CT | 25,119 | 11.551 |  | 8,053 | 0.025 | 327,383 | 11.300 |
| Stage IV |  |  |  |  |  |  |  |
| Routine clinical physical examination | 9,891 | 9.867 |  | — | — | — | 9.768 |
| cfEBV DNA-guided MRI+CT+BS | 12,762 | 9.949 |  | 2,870 | 0.082 | 34,990 | 9.821 |
| cfEBV DNA-guided PET/CT | 14,655 | 9.979 |  | 1,893 | 0.030 | 63,984 | **9.832** |
| Routine MRI+CT+BS | 18,013 | 9.976 |  | 3,358 | -0.002 | Dominated^¶^ | 9.796 |
| Routine PET/CT | 25,837 | 10.021 |  | 11,182 | 0.042 | 264,018 | 9.763 |
| **NCCN Surveillance Protocol** |  |  |  |  |  |  |  |
| Stage I |  |  |  |  |  |  |  |
| Routine clinical physical examination | 8,533 | 13.229 |  | — | — | — | **13.143** |
| cfEBV DNA-guided MRI+CT+BS | 10,321 | 13.240 |  | 1,787 | 0.011 | 159,513 | 13.137 |
| cfEBV DNA-guided PET/CT | 12,152 | 13.243 |  | 1,831 | 0.003 | 565,945 | 13.121 |
| Routine MRI+CT+BS | 17,414 | 13.244 |  | 5,262 | 0.001 | 6,112,352 | 13.070 |
| Routine PET/CT | 26,378 | 13.249 |  | 8,964 | 0.005 | 1,787,576 | 12.985 |
| Stage II |  |  |  |  |  |  |  |
| Routine clinical physical examination | 9,063 | 12.148 |  | — | — | — | 12.057 |
| cfEBV DNA-guided MRI+CT+BS | 11,142 | 12.179 |  | 2,078 | 0.031 | 67,026 | **12.068** |
| cfEBV DNA-guided PET/CT | 13,028 | 12.190 |  | 1,886 | 0.011 | 165,188 | 12.060 |
| Routine MRI+CT+BS | 17,831 | 12.193 |  | 4,803 | 0.003 | 1,725,572 | 12.015 |
| Routine PET/CT | 26,601 | 12.210 |  | 8,770 | 0.017 | 529,068 | 11.944 |
| Stage III |  |  |  |  |  |  |  |
| Routine clinical physical examination | 9,539 | 11.467 |  | — | — | — | 11.372 |
| cfEBV DNA-guided MRI+CT+BS | 11,885 | 11.515 |  | 2,346 | 0.048 | 49,389 | **11.396** |
| cfEBV DNA-guided PET/CT | 13,825 | 11.532 |  | 1,939 | 0.017 | 111,190 | 11.394 |
| Routine MRI+CT+BS | 18,357 | 11.534 |  | 4,532 | 0.002 | 2,494,167 | 11.350 |
| Routine PET/CT | 27,068 | 11.559 |  | 8,711 | 0.025 | 342,971 | 11.289 |
| Stage IV |  |  |  |  |  |  |  |
| Routine clinical physical examination | 10,578 | 9.878 |  | — | — | — | 9.773 |
| cfEBV DNA-guided MRI+CT+BS | 13,489 | 9.960 |  | 2,911 | 0.082 | 35,529 | 9.825 |
| cfEBV DNA-guided PET/CT | 15,551 | 9.993 |  | 2,062 | 0.033 | 63,401 | **9.837** |
| Routine MRI+CT+BS | 19,362 | 9.991 |  | 3,811 | -0.001 | Dominated^¶^ | 9.798 |
| Routine PET/CT | 27,844 | 10.038 |  | 12,293 | 0.045 | 273,332 | 9.759 |

Abbreviations: BS, bone scintigraphy; cfEBV, cell-free Epstein-Barr virus; CT, computed tomography; ICER, incremental cost-effectiveness ratio; MRI, magnetic resonance imaging; NCCN, National Comprehensive Cancer Network; NHB, net health benefit; PET/CT, positron emission tomography/computed tomography; QALY, quality-adjusted life-years; RTOG, Radiation Therapy Oncology Group.

^†^ Routine clinical physical examination consists of history and physical examinations, complete blood counts, comprehensive metabolic panels and nasopharyngoscopies. Other strategies also include routine clinical physical examination. Please see the manuscript for the full description of each strategy.

^‡^ Incremental values were compared with the previous less costly and nondominated strategy.

^§^ Calculated at the willingness-to-pay threshold of $100,000 using the following formula: effectiveness – cost/willingness-to-pay. Strategies with the highest NHB values, highlighted in bold, are considered the most cost-effective.

^¶^ Refer to a strategy that is less effective and more costly than another strategy.

**Additional file 1: Figure S1–S6**

**
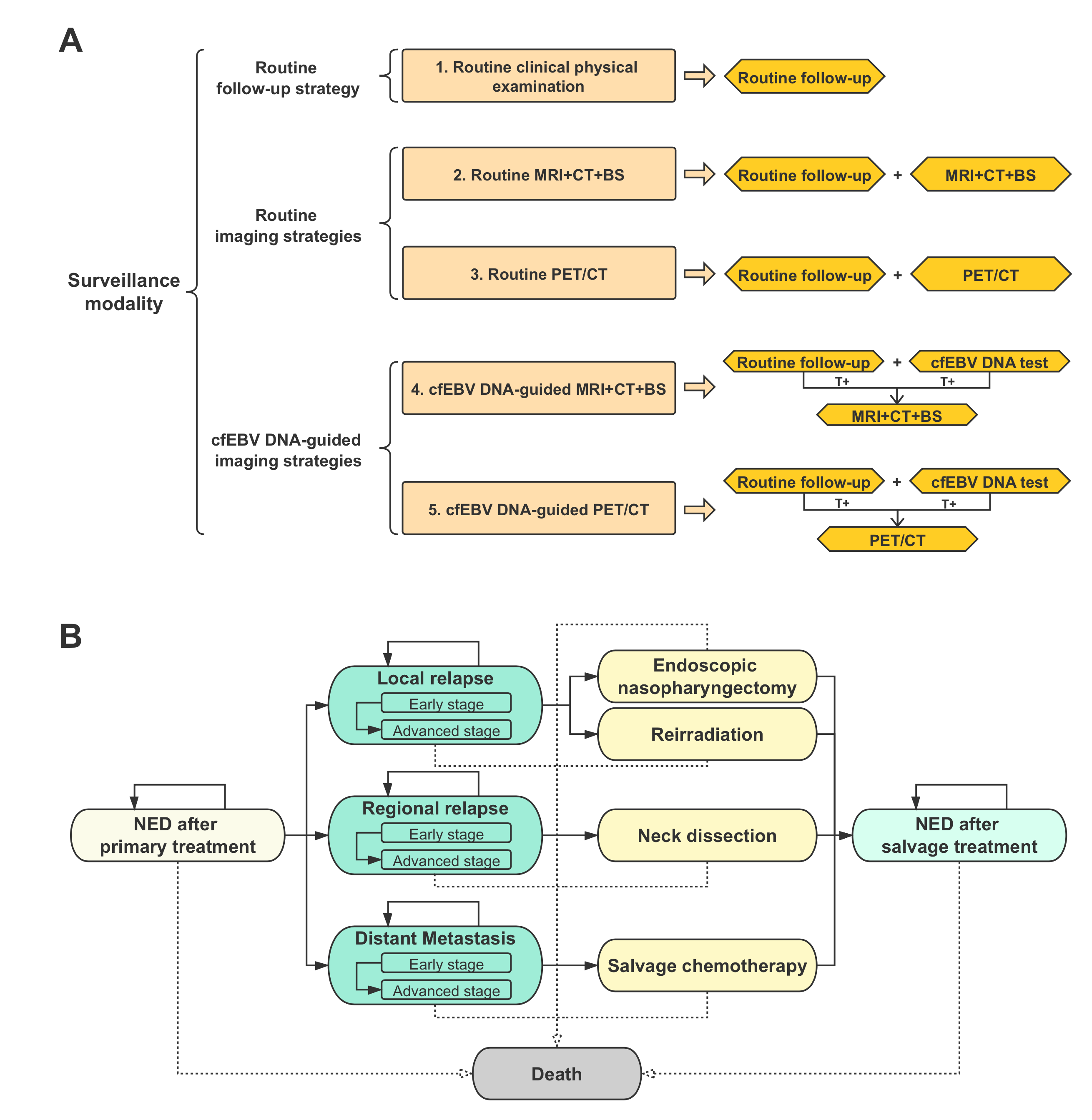
**

**Figure S1.** Schematic diagram of the surveillance strategies and the structure of the Markov model. (A) The five surveillance modalities compared in the study. Boxes with an orange background represent the surveillance tests included in each strategy. The routine follow-up includes history and physical examinations, complete blood counts, comprehensive metabolic panels and nasopharyngoscopies. The routine MRI+CT+BS or PET/CT includes routine follow-up examinations and imaging studies. The cfEBV DNA-guided MRI+CT+BS or PET/CT includes routine follow-up examinations and cfEBV DNA tests, whose positive results would trigger further imaging studies. (B) Markov state transition process, where patients move between the health states based on prespecified monthly transition probabilities. Patients with recurrence can be detected by different surveillance modalities during posttreatment follow-up and receive corresponding treatments, while those undetected will remain in recurrence states or die. Note that patients with early-stage recurrence may progress to advanced-stage recurrence if they remain undetected.

Abbreviations: BS, bone scintigraphy; cfEBV, cell-free Epstein-Barr virus; CT, computed tomography; MRI, magnetic resonance imaging; NED, no evidence of disease; NPC, nasopharyngeal carcinoma; PET/CT, positron emission tomography/computed tomography; T, test.

**
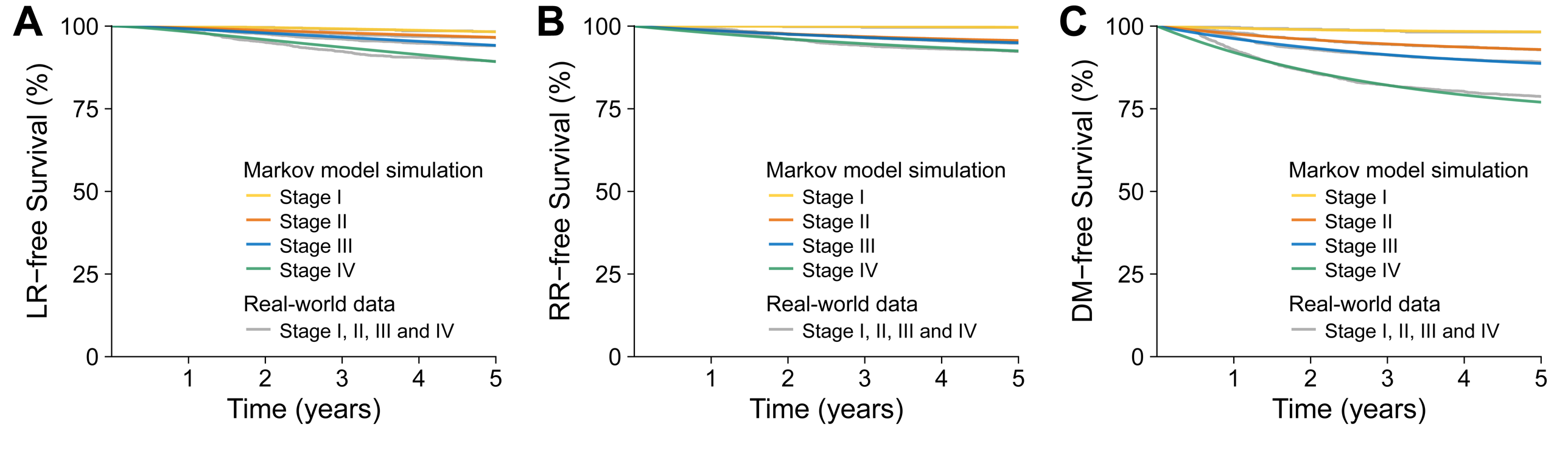
**

**Figure S2.** Validation of the Markov model. The figures demonstrate how the Markov model simulates stage-specific disease recurrence patterns: (A) LR-free survival, (B) RR-free survival and (C) DM-free survival (colored lines) compared with the real-world data (grey lines). The model-simulated survival curves were generated from the Markov model with the base-case model parameters. The real-world survival curves were calculated using the Kaplan-Meier method from the time-to-event data of the NPC cohort containing 10,097 nonmetastatic nasopharyngeal carcinoma patients.

Abbreviations: DM, distant metastasis; LR, local relapse; RR, regional relapse.


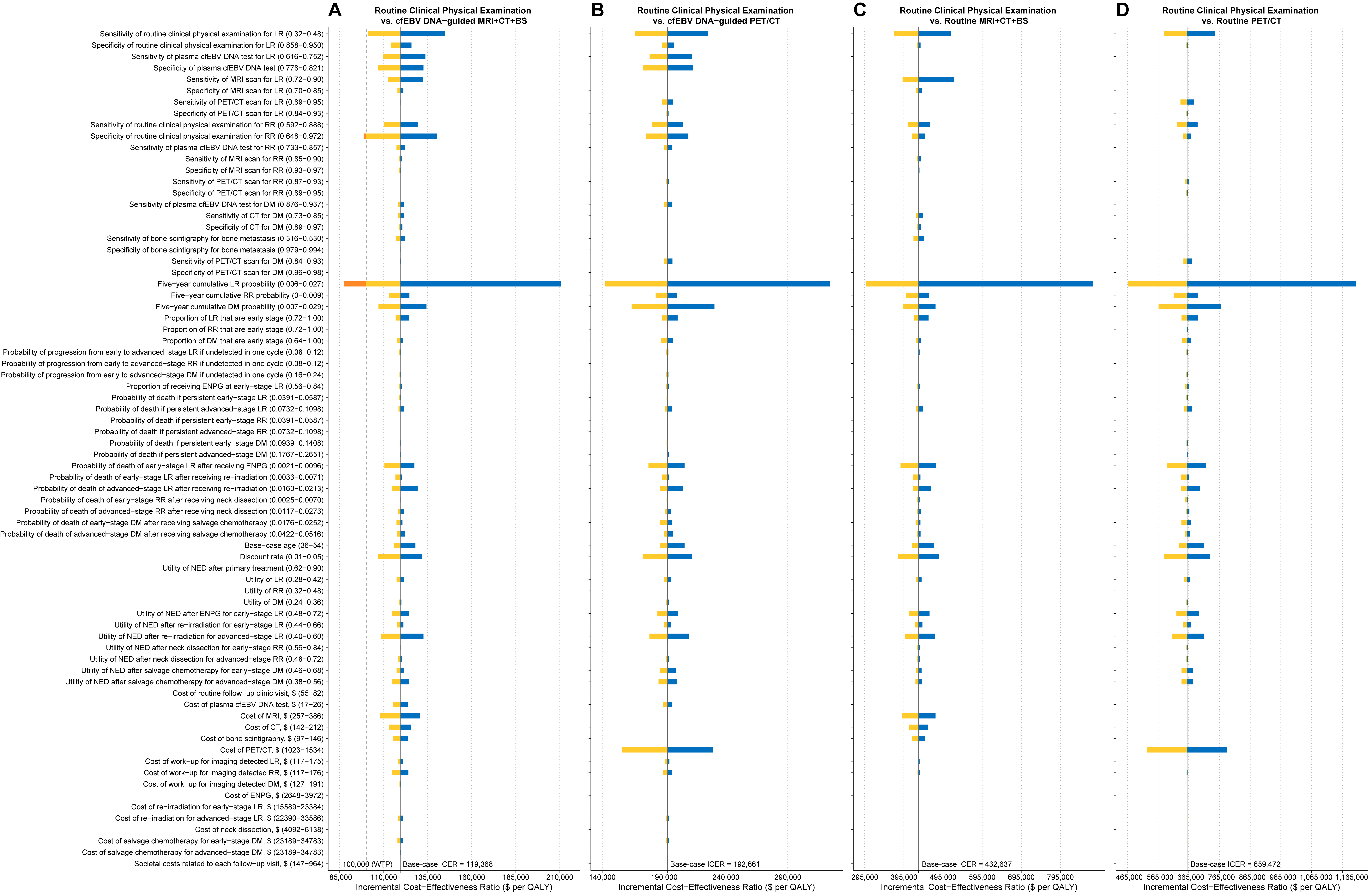


**Figure S3.** Tornado diagram of one-way sensitivity analysis for stage I NPC patients. The figure depicts the influence of the variation of each parameter on the ICERs between routine clinical physical examination and (A) cfEBV DNA-guided MRI+CT+BS; (B) cfEBV DNA-guided PET/CT; (C) routine MRI+CT+BS; (D) routine PET/CT. The blue bars and red bars illustrate the ICERs that are greater and less than the base-case ICERs, respectively. The orange bars indicate that the ICERs go across the willingness-to-pay threshold, leading to a switch of the most cost-effective strategy. The range of each parameter utilized in the sensitivity analysis was displayed in parentheses on the y-axis. The solid and dashed lines represent the ICERs in the base-case analysis and the willingness-to-pay threshold of $100,000 per quality-adjusted life-year, respectively.

Abbreviations: BS, bone scintigraphy; cfEBV, cell-free Epstein-Barr virus; CT, computed tomography; DM, distant metastasis; ENPG, endoscopic nasopharyngectomy; ICER, incremental cost-effectiveness ratio; LR, local relapse; MRI, magnetic resonance imaging; NED, no evidence of disease; NPC, nasopharyngeal carcinoma; PET/CT, positron emission tomography/computed tomography; RR, regional relapse; WTP, willingness-to-pay.


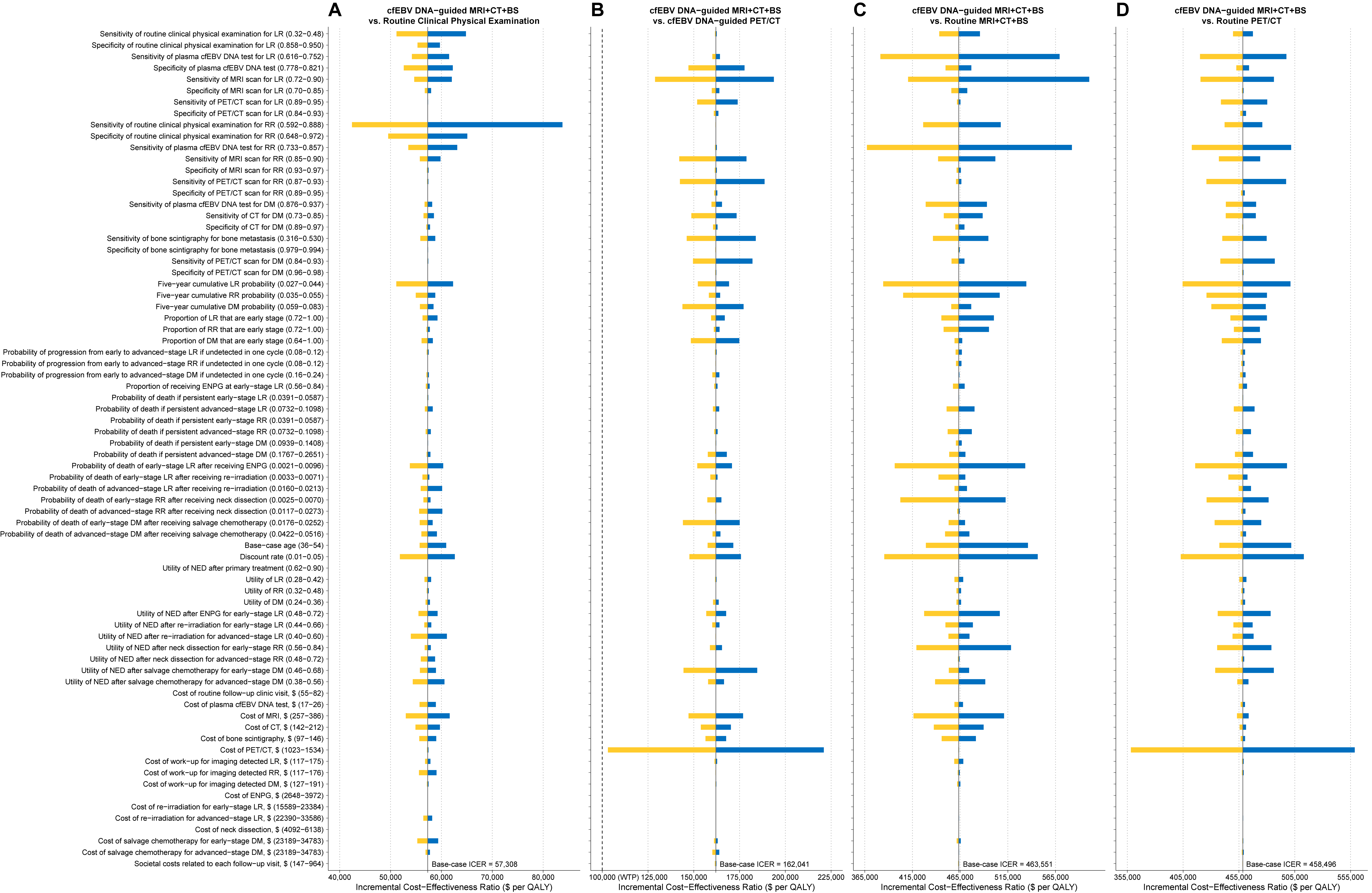


**Figure S4.** Tornado diagram of one-way sensitivity analysis for stage II NPC patients. The figure depicts the influence of the variation of each parameter on the ICERs between cfEBV DNA-guided MRI+CT+BS and (A) routine clinical physical examination; (B) cfEBV DNA-guided PET/CT; (C) routine MRI+CT+BS; (D) routine PET/CT. The blue bars and red bars illustrate the ICERs that are greater and less than the base-case ICERs, respectively. The range of each parameter utilized in the sensitivity analysis was displayed in parentheses on the y-axis. The solid and dashed lines represent the ICERs in the base-case analysis and the willingness-to-pay threshold of $100,000 per quality-adjusted life-year, respectively.

Abbreviations: BS, bone scintigraphy; cfEBV, cell-free Epstein-Barr virus; CT, computed tomography; DM, distant metastasis; ENPG, endoscopic nasopharyngectomy; ICER, incremental cost-effectiveness ratio; LR, local relapse; MRI, magnetic resonance imaging; NED, no evidence of disease; NPC, nasopharyngeal carcinoma; PET/CT, positron emission tomography/computed tomography; RR, regional relapse; WTP, willingness-to-pay.


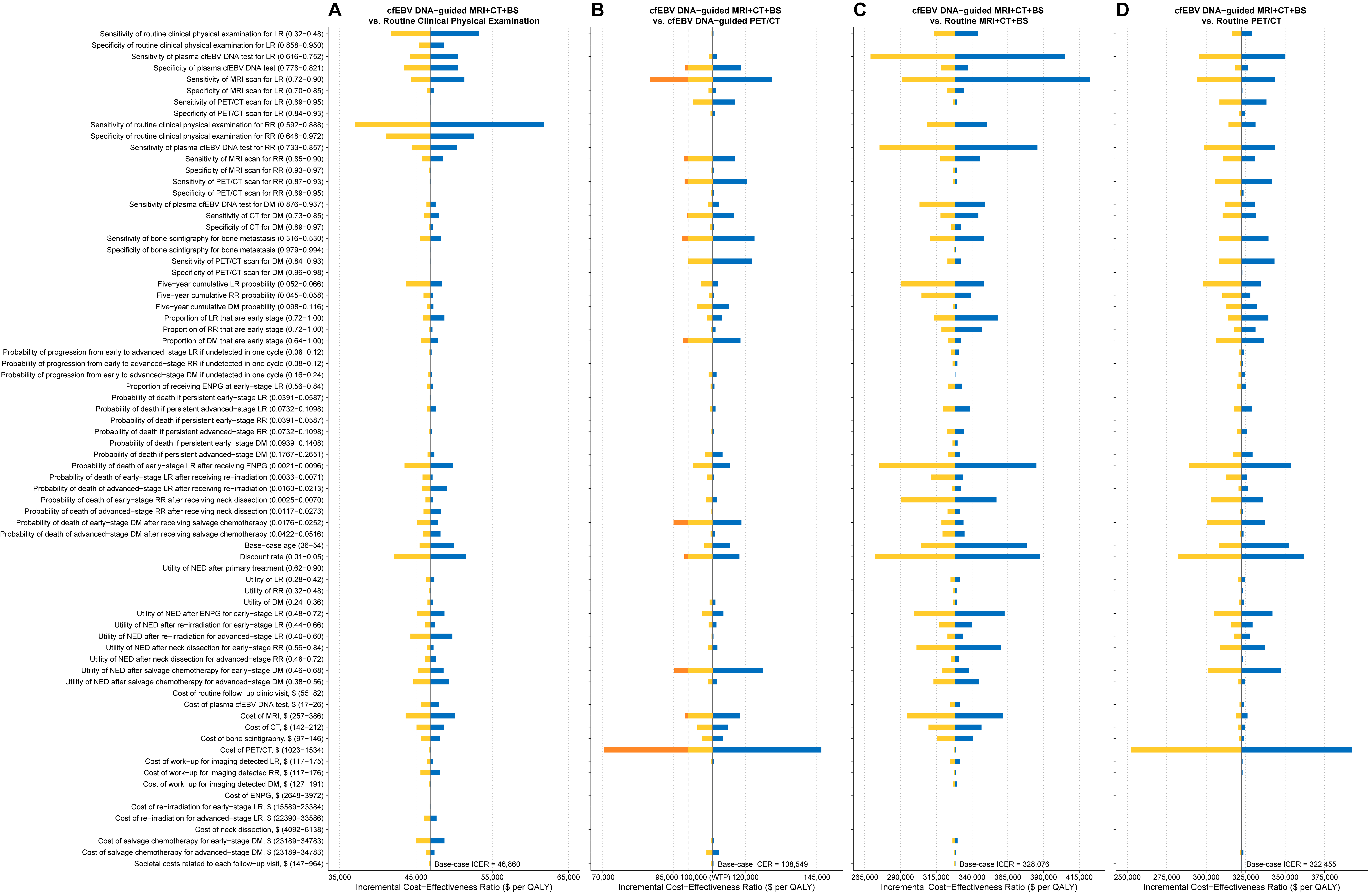


**Figure S5.** Tornado diagram of one-way sensitivity analysis for stage III NPC patients. The figure depicts the influence of the variation of each parameter on the ICERs between cfEBV DNA-guided MRI+CT+BS and (A) routine clinical physical examination; (B) cfEBV DNA-guided PET/CT; (C) routine MRI+CT+BS; (D) routine PET/CT. The blue bars and red bars illustrate the ICERs that are greater and less than the base-case ICERs, respectively. The orange bars indicate that the ICERs go across the willingness-to-pay threshold, leading to a switch of the most cost-effective strategy. The range of each parameter utilized in the sensitivity analysis was displayed in parentheses on the y-axis. The solid and dashed lines represent the ICERs in the base-case analysis and the willingness-to-pay threshold of $100,000 per quality-adjusted life-year, respectively.

Abbreviations: BS, bone scintigraphy; cfEBV, cell-free Epstein-Barr virus; CT, computed tomography; DM, distant metastasis; ENPG, endoscopic nasopharyngectomy; ICER, incremental cost-effectiveness ratio; LR, local relapse; MRI, magnetic resonance imaging; NED, no evidence of disease; NPC, nasopharyngeal carcinoma; PET/CT, positron emission tomography/computed tomography; RR, regional relapse; WTP, willingness-to-pay.


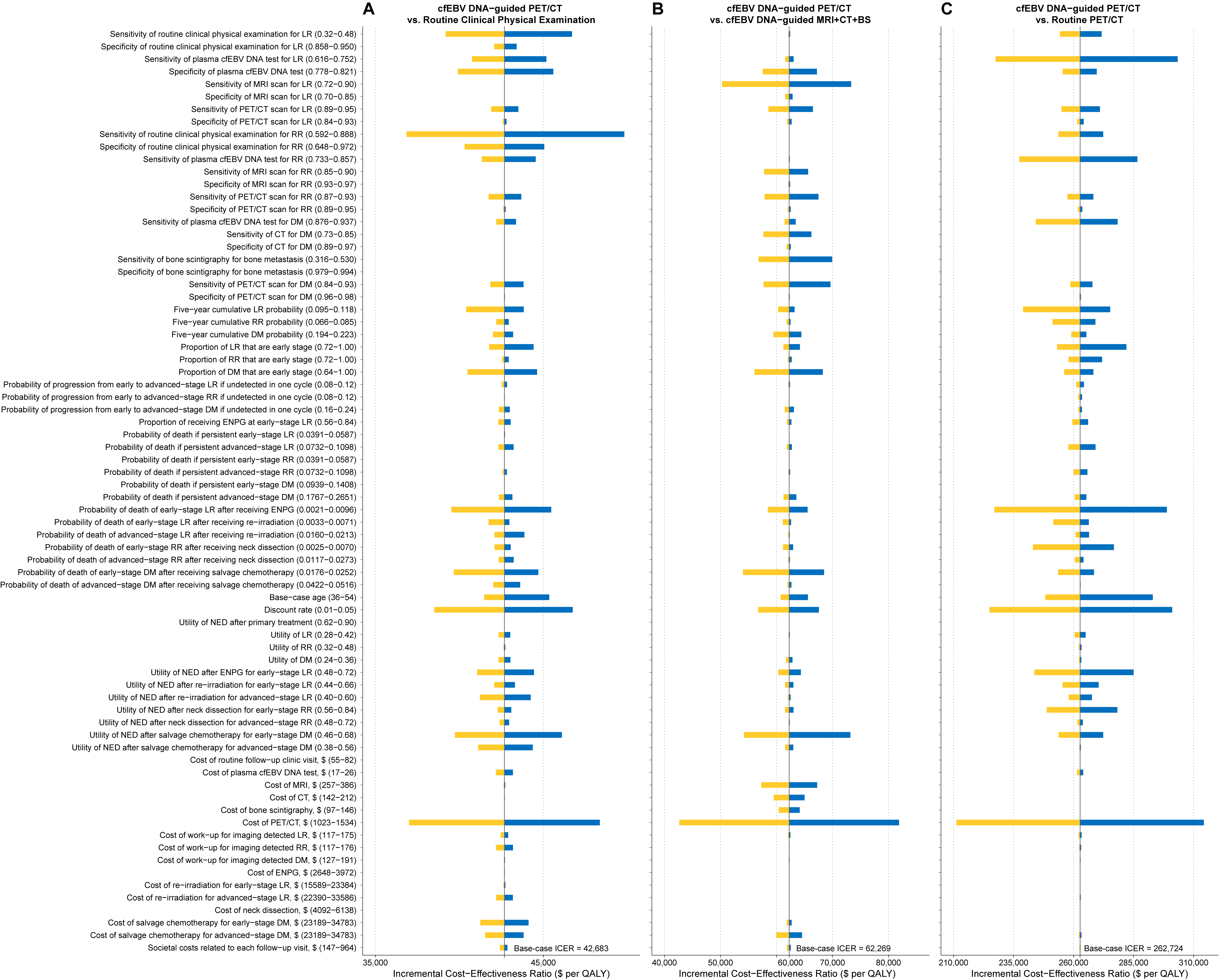


**Figure S6.** Tornado diagram of one-way sensitivity analysis for stage IV NPC patients. The figure depicts the influence of the variation of each parameter on the ICERs between cfEBV DNA-guided PET/CT and (A) routine clinical physical examination; (B) cfEBV DNA-guided MRI+CT+BS; (C) routine PET/CT. The tornado diagram of one-way sensitivity analysis between cfEBV DNA-guided PET/CT and routine MRI+CT+BS is not shown because the ICERs are dominated for all values. The blue bars and red bars illustrate the ICERs that are greater and less than the base-case ICERs, respectively. The range of each parameter utilized in the sensitivity analysis was displayed in parentheses on the y-axis. The solid and dashed lines represent the ICERs in the base-case analysis and the willingness-to-pay threshold of $100,000 per quality-adjusted life-year, respectively.

Abbreviations: BS, bone scintigraphy; cfEBV, cell-free Epstein-Barr virus; CT, computed tomography; DM, distant metastasis; ENPG, endoscopic nasopharyngectomy; ICER, incremental cost-effectiveness ratio; LR, local relapse; MRI, magnetic resonance imaging; NED, no evidence of disease; NPC, nasopharyngeal carcinoma; PET/CT, positron emission tomography/computed tomography; RR, regional relapse; WTP, willingness-to-pay.
