# Supplementary material for: Liquid biopsy posttreatment surveillance in endemic nasopharyngeal carcinoma: a cost-effective strategy to integrate circulating cell-free Epstein-Barr virus DNA
Source: BMC Med. 2021 Aug 26;19:193. doi: 10.1186/s12916-021-02076-4 (PMC8390246; doi:10.1186/s12916-021-02076-4)
Supplement: Supplementary file 2 — Additional file 2. Supplementary methods. [file 12916_2021_2076_MOESM2_ESM.docx]

**Additional File 2:**

**Liquid Biopsy Posttreatment Surveillance in Endemic Nasopharyngeal Carcinoma: A Cost-Effective Strategy to Integrate Circulating Cell-Free Epstein-Barr Virus DNA**

**Additional file 2: Supplementary Methods**

***Information of the NPC Cohort of 10,097 Patients***

*Data Extraction*

The nasopharyngeal carcinoma (NPC)-specific database from the Big Data Intelligence Platform of Sun Yat-sen University Cancer Center (Guangzhou, China) is a patient-level research system that enables the real-time organization, integration and updating of medical records automatically from several clinical business systems based on well-designed data models and algorithms. It allows users to set query conditions to search for matched cases, visualize data, and enroll eligible cases into retrospective or prospective cohorts. Details of this platform were described in our previous study [38].

From the NPC-specific database, we retrieved the data of 10,097 patients with nonmetastatic, biopsy-proven NPC diagnosed between April 2009 and December 2015 at Sun Yat-sen University Cancer Center. Patients were included if they achieved complete remission (CR) after radical treatment for NPC. According to the Response Evaluation Criteria in Solid Tumors 1.1, CR was defined as no unequivocal soft tissue mass in the local region and all lymph nodes less than 10 mm in the short axis under head and neck magnetic resonance imaging (MRI) and flexible nasopharyngoscopy 12–16 weeks after radiotherapy [57]. Based on the patient-level time-to-event data of the 10,097 patients, monthly probabilities of local relapse (LR), regional relapse (RR) and distant metastasis (DM) were calculated by parametric survival models using flexsurv package in R. The cohort's authenticity has been validated by uploading the key raw data onto the research data deposit (RDD) public platform (http://www.researchdata.org.cn, approval RDD number: RDDA2021001822) [58].

The median follow-up time of the cohort was 67.3 months (interquartile range, 54.0–83.0 months). In the study population, a total of 705 (6.98%) patients developed LR, 553 (5.48%) patients developed RR and 1,307 (12.9%) patients developed DM. Detailed patient characteristics are presented in Additional file 1: Table S6.

*Diagnostic Work-up, Staging and Follow-up*

Routine pre-treatment evaluations included: detailed patient history, physical examinations, hematology and biochemistry profiles, plasma circulating cell-free Epstein-Barr virus (cfEBV) DNA test, fiberoptic nasopharyngoscopy, MRI of the head and neck, chest radiography, abdominal sonography, whole-body bone scintigraphy or contrast-enhanced chest and abdomen computed tomography (CT) or 18F-fluorodeoxyglucose positron emission tomography/computed tomography (PET/CT).

All patients were restaged according to the Union for International Cancer Control/American Joint Committee on Cancer Eighth edition staging system [59]. Restaging was independently performed by two senior radiation oncologists who specialized in head and neck cancers, and disagreements were resolved by consensus.

Patients were regularly followed up every 3 months during the first 2 years, every 6 months during the 3 to 5 years, and annually thereafter. At each visit, detailed patient history, physical examinations, plasma cfEBV DNA test and fiberoptic nasopharyngoscopy were routinely performed. MRI of the head and neck, chest radiography, abdominal sonography, bone scan or CT or PET/CT were repeated annually or when clinically suspected recurrence occurred. The follow-up duration was calculated from the date of diagnosis to either death or last follow-up. The date of last follow-up was October 2019.

*Treatment*

All patients received radical intensity-modulated radiotherapy (IMRT) with or without chemotherapy as primary treatment for NPC. During the study period, our institutional guidelines recommended IMRT alone for stage I patients and platinum-based concurrent chemoradiotherapy with or without induction/adjuvant chemotherapy for stage II–IVA patients. Reasons for deviation of treatment from our institutional guidelines included age, patient's refusal of treatment, or organ dysfunction suggestive of intolerance to treatment. Salvage treatments, including reirradiation, chemotherapy, and surgery, were provided during recurrence or persistent disease.

The nasopharyngeal and neck tumor volumes were treated for the entire treatment course. Tumor target volumes were delineated according to our institutional guidelines, in accordance with the International Commission on Radiation Units and Measurements reports 62 and 83 [60, 61]. The prescribed doses were 66–72 Gy/28–33 fractions to the planning target volume (PTV) of the primary gross tumor volume (GTVnx), 64–70 Gy/28–33 fractions to the PTV of the GTV of the involved lymph nodes (GTVnd), 60–63 Gy/28–33 fractions to the PTV of the high-risk clinical target volume (CTV1), and 54–56 Gy/28–33 fractions to the PTV of the low-risk clinical target volume (CTV2). All patients were treated using the IMRT with the simultaneous integrated boost technique, following a routine schedule of one fraction daily, 5 days per week for a total of 6–7 weeks. Details about the IMRT technique used at our center were described previously [62].

The induction chemotherapy regimens included two to four cycles of 3-weekly TPF regimens (cisplatin 75 mg/m^2^ on day 1, docetaxel 75 mg/m^2^ on day 1, and 5-flurouracil 500 mg/m^2^/d continuous intravenous infusion on day 1–5), TP regimens (docetaxel 75 mg/m^2^ on day 1 and cisplatin 75 mg/m^2^ on day 1), PF regimens (cisplatin 75 mg/m^2^ on day 1 and 5-FU 500 mg/m^2^/d continuous intravenous infusion on day 1–5) and GP regimens (cisplatin 80 mg/m^2^ on day 1 and gemcitabine 1,000 mg/m^2^ on day 1 and day 8).

The concurrent chemotherapy regimens, beginning on the first day of radiotherapy, comprised of platinum (mainly cisplatin or nedaplatin) 30–40 mg/m^2^ every week for five to six cycles, 80 mg/m^2^ every 3 weeks for two to three cycles or 100 mg/m^2^ every 3 weeks for two to three cycles.

The adjuvant chemotherapy regimens mainly included two to four cycles of 3-weekly TP regimens (docetaxel 75 mg/m^2^ on day 1 and cisplatin 75 mg/m^2^ on day 1) and PF regimens (cisplatin 75 mg/m^2^ on day 1 and 5-FU 500 mg/m^2^/d continuous intravenous infusion on day 1–5).

***Literature Search and Data Extraction***

The majority of the model parameters were derived from published studies. To retrieve the parameter data, we comprehensively searched PubMed and EMBASE for suitable references from January 1990 to October 2020 without language restrictions. Summarized data from systematic reviews or meta-analyses were considered the evidence with the highest quality, followed by data from randomized controlled trials or retrospective studies. If more than one reference was eligible, those with larger sample sizes were regarded as having higher quality.

Probabilities, clinical utilities and costs were extracted from the selected studies. If survival probabilities were not recorded directly in textual form in the literature, WebPlotDigitizer software (version 4.3, https://automeris.io/WebPlotDigitizer) was employed to extract the probabilities from the survival curves, where three investigators independently extracted the data and then averaged them, with any disagreements resolved by consensus. If more than one cohort in each category was included, the weighted average of survival probabilities based on the number of patients in each cohort was calculated.
